# Physical activity experiences among children with ADHD and ASD: a qualitative meta-synthesis literature review

**DOI:** 10.1080/17482631.2025.2524460

**Published:** 2025-07-23

**Authors:** Karin Grahn

**Affiliations:** Department of Food and Nutrition and Sport Science, University of Gothenburg, Gothenburg, Sweden

**Keywords:** Disability, attention deficit hyperactivity disorder, autism spectrum disorder, sport, physical education, socio ecological model, qualitative research

## Abstract

**Purpose:**

The aim was to analyze and synthesize empirical research on physical activity experiences among children diagnosed with ADHD and ASD.

**Methods:**

A qualitative meta-synthesis was conducted, including 17 articles published between 2010 and 2023. The synthesis encompasses qualitative research on the experiences of children with ADHD and ASD in organized physical activity. Data were categorized into overarching themes based on a socio-ecological model. Each overarching theme was subdivided into themes and sub-themes illustrated with extracts from each study.

**Results:**

The research was conducted in several countries, with a predominance of studies from the USA and Canada. Most studies focused on children diagnosed with ASD, with boys being more frequently represented. Various intrapersonal factors were influential, with negative factors including disability-specific constraints, motor skill difficulties, and dissatisfaction with physical activity, while enjoyment of specific activities and positive attitudes towards physical activity were identified as some of the positive factors. In terms of interpersonal factors, interactions with family, friends, teammates, and coaches or teachers shaped both positive and negative experiences.

**Conclusions:**

The findings provide valuable insights into the experiences of children with ADHD and ASD in organized physical activity contexts. Key aspects identified can guide future research and initiatives aimed at including children with ADHD and ASD in organized physical activities.

## Introduction

Attention deficit hyperactivity disorder (ADHD) and autism spectrum disorder (ASD) are neurodevelopmental disabilities leading to impairments influencing everyday life.^1^ ADHD affects children’s attention, impulse control, and activity levels; while ASD consists of impairment in social interaction, communication, and behavioural rigidity (American Psychiatric Association, 2013). ADHD and ASD have also been shown to interrelate with poor movement skills (Green et al., 2009; Harvey et al., 2014). One manifestation of these conditions is their impact on their engagement with and experience of physical activity. Children diagnosed with ADHD and ASD are generally less physically active, less likely to meet the recommended physical activity levels and have more negative experiences of organized forms of physical activity than their age-related peers (e.g., Ayvazoglu et al., 2015; Harvey et al., 2009, 2014; Johnson & Rosen, 2000; Kim et al., 2011; Quesada et al., 2018; Stanish et al., 2015). They tend to drop out of sports early (Johnson & Rosen, 2000) and spend more time doing sedentary activities (Tandon et al., 2019). Lack of physical activity has a major negative impact on both physical and mental health and wellbeing, and therefore the fact that these groups are not engaging is a very important issue to understand and address.

Children’s^2^ engagement in physical activity is significantly influenced by their experiences in various settings, such as physical education (PE), sports, and community-based physical activity programmes. Reasons why children diagnosed with ADHD and ASD tend to be less physically active are complex and affected by several different factors, such as safety issues, difficulties with social interaction, being excluded, lack of structure and predictability, lack of enjoyment or self-doubt (Brewster & Coleyshaw, 2011; Coates & Vickerman, 2008, 2010; Shimoni et al., 2010). Previous research by Harvey et al. (2009) showed that boys with ADHD had fewer movement skills than age-related peers, had superficial knowledge about how to preform-specific movements, and had a more negative experience of physical activity. Additionally, a questionnaire by Stanish et al. (2015) indicated that adolescents with ASD enjoyed PE and team sports less and participated in leisure-time sports less frequently compared to their non-diagnosed peers. They also found physical activity hard to learn and did not view organized physical activity as a means of fostering friendships. Furthermore, according to parental reports, safety or unsafety in the surrounding (Blagrave & Colombo-Dougovito, 2019; Gürkan et al., 2023; Hickingbotham et al., 2021; Siu & Lo, 2020) or lack of adapted suitable physical activity programs (Arnell et al., 2020; Nichols et al., 2019; Papadopoulos et al., 2020) are other factors influencing children’s participation.

Understanding what affects children’s physical activity experiences, from the micro level (e.g., lack of motor ability) to the macro level (e.g., participation opportunities), is crucial for designing health-promoting interventions for children with ADHD and ASD (cf. McLeroy et al., 1988). In the effort to advance knowledge on disabled children’s participation in physical activities, scholars have noted a lack of studies on *children’s own experiences* (eg. Fitzgerald et al., 2003; Harvey et al., 2009). However, in the last 10–15 years, more child-centred research has emerged to highlight *children’s perspectives*. Two previous literature reviews on the subject were conducted with children with physical disabilities (Coates & Vickerman, 2008) and students with diverse conditions such as health-related illness, learning disabilities, ASD and sensory disabilities (Haegele & Sutherland, 2015). Both focused on engagement with school-based PE. However, whilst these reviews add important knowledge to the field, they are limited to PE and do not include children with ADHD or have a specific focus on ASD. Moreover, there remains a shortage of synthesized knowledge regarding the physical activity experiences of children with neurodevelopmental disorders. Previous studies demonstrate a significant gap in research, particularly in children’s perspectives. Given the consequences of a lack of physical activity for both physical and mental well-being, particularly for groups that already face several disadvantages and limitations, it is crucial to add a new contribution to existing knowledge. Specifically, there is a need for research involving experiences of children with ADHD and ASD participating in organized physical activity. The purpose of this study is to carry out a meta-synthesis review of qualitative research to analyse and synthesize empirical research on physical activity experiences among children diagnosed with ADHD and ASD. This will be done by (a) creating an overview of previous research; (b) synthesizing how previous research describes experiences of organized physical activity among children with ADHD and ASD within the framework of a socio-ecological model; and (c) synthesizing positive and negative experiences that are presented in previous research. Before displaying the synthesized result, the theoretical framework and methodological underpinnings will be presented.

## Theoretical and methodological framework

Whilst analyzing previous research, it became clear that experiences of organized physical activity among children diagnosed with ADHD or ASD are affected by several factors on diverse levels, from individual factors to societal factors. Inspired by McLeroy et al. (1988)’s framework on diverse factors influencing an individual’s health behaviours, I have applied these insights to understand how children’s positive and negative experiences in organized physical activity are shaped not solely by their disability, but also by interactions with others and the broader societal context. McLeroy et al. (1988) articulate several levels influencing individual health behaviours within internal and external forces affecting the individual. These can be explained as: *intrapersonal*, *interpersonal*, *institutional*, *community* and *public policy* factors. *Intrapersonal factors* influencing physical activity experiences can include loss of focus, poor movement skills or anxiety, while *interpersonal factors* are related to social relations such as influence by peers or coaches or teachers. *Institutional factors* are tied to the context of specific institutions such as schools or sport clubs. *Community factors* include the opportunity to join sports programs or adapted sports programs. Finally, *public policy factors* may affect a child’s participation experiences through the application of rules and regulations, for example, the integration of children with neurodevelopmental disorders within mainstream PE (Thoren et al., 2021). Besides McLeroy’s five levels, Sallis et al. (1998) argue for adding *physical/environmental factors* since the physical environment is essential for physical education participation. These may be equipment or clothes used in the activity, or external factors such as light, temperature and weather. In the analysis, the socioecological model is used, as a synthesizing tool, to sort diverse factors influencing children’s experiences.

Further, to enable the child’s perspective to come to the foreground, the paper takes its point of departure in the importance of emphasizing children’s perspectives and treating their voices as legitimate study subjects (Fargas-Malet et al., 2010; James, 1998). Therefore, articles focusing on children’s own experiences are in the foreground in this review.

## Material and methods

A qualitative meta-synthesis literature review was conducted (Sandelowski, 2007). This review type is suitable when one wants to retrieve and review results from previous qualitative findings into a synthesized result that generates greater understanding than simply being the sum of each individual research report. Qualitative meta-synthesis includes an interpretative framework and depart from a socio-constructivist paradigm. The reason for synthesizing results from *qualitative studies* is based on the interest in children’s experiences. Qualitative research is a suitable technique to use to explore this.

This meta synthesis offers an integrated description of previous qualitative reports on children with ADHD and ASD and their experiences of participating in organized physical activity. The method includes three steps: 1) a systematic search of qualitative research on the topic; 2) a critical appraisal of the research found according to inclusion criteria; and 3) an interpretative integration of the findings, creating themes and sub-themes (Sandelowski, 2007). These three steps are each described in more detail below.

### Step 1: systematic search and retrieval of empirical studies

**Figure 1.**
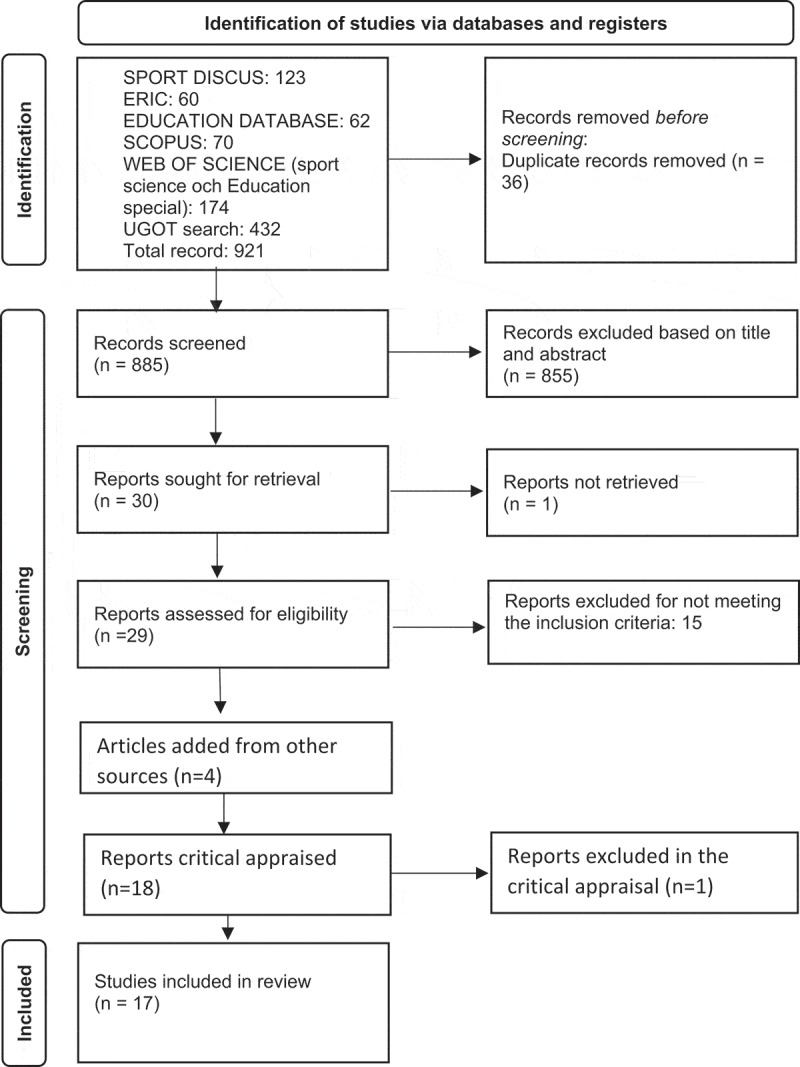
Modified PRISMA flow diagram.

A comprehensive search strategi was used. The literature was searched for in six databases (see Figure 1). These databases were selected to include a mix of sport science, social science and educational research. The search process was conducted in consultation with a librarian who gave feedback on search terms, databases and strategies for searching.

This work is licenced under CC BY 4.0. To view a copy of this licence, visit https://creativecommons.org/licenses/by/4.0/

Search words were decided on by using PICo for qualitative studies, including population, phenomenon of interest and context. The *population* was determined as being children and/or adolescents diagnosed with ADHD or ASD. *Interest* was defined as experiences of organized physical activity. Finally, the *context* was limited to organized physical activity, i.e., physical activities that are organized and led by a sport coach, leader or PE teacher. This includes, PE, adapted PE, self-contained PE, adapted sports,^3^ competitive sports and other leader-led physical activities such as community-based physical activity programmes.

The sample was limited to peer-reviewed articles, written in English and published between 2010 and 2021, which was later expanded with literature from 2022 to 2023.^4^ Starting the search 2010 is motivated by the last 10–15 years of a growing body of research that gave greater attention to children’s voices regarding their experiences in physical activities. The extension of literature was done since these later documents became available after the study had begun.

### Step 2: a critical appraisal of empirical studies

Inclusion criteria were articles on (1) children diagnosed with ADHD or ASD; (2) context of organized physical activity; (3) focus on children’s experiences; (4) empirical studies; (5) qualitative research; and (6) articles written in English. Articles were excluded if they covered adults’ experiences. Further, research on any organized physical activity that made no mention of ADHD, ASD or hidden disability^5^ were excluded. Studies on leisure time activities without specifying organized physical activity were also excluded. Articles aiming to evaluate the effect of physical activity as a treatment for ADHD/ASD symptoms were not included, these studies aim to explore the effect of the treatment not the experience of the activity.

Articles were first scanned on titles and second on abstracts to determine relevance. A high proportion of the articles were considered non-relevant. This was due to the search process brought up literature on physical activity and children with ADHD or ASD but few of these studies explored *experiences* from the *child’s perspective*. Articles that were deemed relevant were exported to the reference system End Note and checked for duplicates. Articles were then read in full, and any remaining articles not matching the inclusion criteria were excluded. These included articles concerning parent’s perceptions of physical activity participation for children with ADHD and ASD from a parental perspective. A few articles found elsewhere, e.g., references from included articles, were added to the sample. Even though I searched specifically for articles portraying children’s own voices, few articles were found. Two articles were retrospective studies with a focus on childhood experiences, these were included. In total, 18 articles were identified as relevant and were included for a critical appraisal using the JBI Checklist for Qualitative Research (JBI, 2020).

Each article was read and appraised by the main author, both individually and collectively. The JBI checklist (JBI, 2020) was used for the individual appraisal. This tool comprises ten questions posed while reading the text, evaluating the congruity between, for example, the methodological perspective and the methods used, as well as the analysis, interpretation, and conclusions drawn. Further aspects, such as researcher influence and ethical considerations, are also taken into account (see further JBI, 2020). After the individual appraisal, one article (Coates & Vickerman, 2010) was excluded as the main findings were based on survey data, and the supplementary focus group interview included only one child with ADHD. It was considered difficult to conclude relevance to this specific synthesis based on the qualitative parts of this study. The remaining articles were deemed relevant and of satisfactory quality.

To enable a collectively appraisal, each article was read and summarized in a table containing information on author, publication year, title, aim, type of PA, country, sample, method (incl. data generation and analysis), main result, and conclusion. This offered an overview of all articles to be used for further analysis. All included studies had methods, analysis and findings that were relevant for the current literature review. All studies used qualitative methods some was included in a mixed method design (Boucher et al., 2023; Harvey et al., 2014; Luymes et al., 2022; Obrusnikova & Cavalier, 2011). In these, the qualitative part was included in the synthesis of results. Several articles used child-centred approaches. After the appraisal of each individual study and the collectively appraisal, 17 articles were included in the third step of analyses, i.e., synthesizing results. The search process, including the critical appraisal of studies, is visualized in Figure 1, a modified PRISMA flow diagram (Page et al., 2021).

### Step 3: synthesizing previous research

To answer the first research question, a table was constructed showing characteristics on included studies, such as type of organized physical activity (LTPA or PE), country of research, included research participants and the perspective of the study subjects (retrospective accounts of childhood or contemporary child perspective (Table 1).

**Table 1.** Information on characteristics of included studies.

No	Study	Country	Type of org. PA^7^	Age	Gender	ADHD or ASD Diagnosis	Other INformation	Perspective	Data production & analysis
1	Arkesteyn et al. (2023)	Belgium	LTPA (sports) PE	12–18	11 boys, 6 girls	ASD	IQ > 70, proficient knowledge of Dutch	Contemporary child perspectives	Semi-structured interviews. Content analysis.
2	Arnell et al. (2018)	Sweden	LTPA PE	12–17	7 girls, 17 boys	ASD	No intellectual disability	Contemporary child perspectives	Qualitative interviews. NVivo. Inductive qualitative latent content analysis.
3	Blagrave (2017)	USA	APE	10–14	1 girl, 9 boys	ASD	Ability to communicate verbally (some with support)	Contemporary child perspectives	Phenomenological approach. Drawings, observation, semi-structured interview. Thematic analysis.
4	Boucher et al. (2023)	Canada	LTPA	8–16	13 boys, one girl	ASD + intellectual disability	IQ between 53–70, English speaking	Contemporary child perspectives	Semi-structured interviews Descriptive phenomenological approach.
5	Haegele and Maher (2022)	USA	PE	13–18	8 boys	ASD	Ability to communicate verbally	Contemporary child perspectives	Semi-structured interviews. Reflexive thematic analysis.
6	Haegele and Maher (2023)	USA	PE	13–18	8 boys	ASD	Ability to communicate verbally	Contemporary child perspectives	Semi-structured interviews. Thematic analysis. Creative non-fiction story.
7	Harvey et al. (2012)	Canada	LTPA	9–12	2 girls, 4 boys	ADHD + ODD, CD, SA^8^	Mother tongue English	Contemporary child perspectives	Scrapbook interviewing (pilot study). Thematic analysis.
8	Harvey et al. (2014)	Canada	LTPA	9–11	2 girls, 8 boys	ADHD	IQ > 70, English speaking	Contemporary child perspectives	Scrapbook interviewing. Thematic analysis.
9	Healy et al. (2013)	Ireland	PE	9–13	1 girl, 11 boys	ASD		Contemporary child perspectives	Semi-structured interviews. Thematic analysis.
10	Ing and Mills (2019)	UK	LTPA (team sport)	10 years ->	1 male	ADHD		Retrospective accounts of childhood	Auto ethnography. Personal and academic voice framework.
11	Jachyra et al. (2021)	Canada	LTPA	12–18	10 boys	ASD	IQ > 70 (*n* = 8), IQ < 70 (*n* = 2)	Contemporary child perspectives	Digital stories and two semi-structured interviews. Thematic analysis.
12	Lamb et al. (2016)	England	PE	11–16	1 girl, 4 boys	ASD + ADHD (*n* = 1)		Contemporary child perspectives	Photo-elicitation unstructured interviews. Thematic analysis.
13	Lee et al. (2014)	Canada	LTPA (team sport)	5–18	6 boys	ADHD		Retrospective accounts of childhood	Semi-structured interviews. Interpretative phenomenological analysis.
14	Luymes et al. (2022)	Canada	LTPA (PA program)	7	One girl	ASD+ADHD, speech disorder, anxiety		Contemporary child perspectives	Video recordings, observations, interviews Content analysis.
15	Obrusnikova and Cavalier (2011)	USA	LTPA (After school PA)	8–14	2 girls, 12 boys	Autism (*n* = 1) Asberger (*n* = 10) Pervasive Development Disorder (*n* = 1)	Verbal skills to communicate, ability to use a digital camera	Contemporary child perspectives	Photo voice & semi-structured interviews. NVivo.
16	Pellerin et al. (2022)	USA	Self-contained PE	7–20	4 girls (one with ASD), 16 boys	ASD (*n* = 12), other congenital disabilities (*n* = 8)	Language skills to participate in interviews	Contemporary child perspectives	Semi-structured interviews, drawings, researcher notes. Inductive thematic analysis.
17	Yessick et al. (2020)	USA	Self- contained PE	11–12	4 boys	ASD	Language skills to participate in interviews	Contemporary child perspectives	Electronic scrapbook, semi- structured interviews. Observational field work. Thematic analysis.

To answer the second and third research questions, the synthesis was conducted through thematic analysis of the data. First, in a deductive process, each included article was read carefully and data extracts from the results sections were sorted by the first author into pre-determined overarching themes based on a socioecological model (McLeroy et al., 1988; Sallis et al., 1998). A data-handling file was used to keep track of the various steps leading to the synthesis of results. Second, in an inductive process, data with similar content were grouped and labelled with key words. Key words representing similar experiences were then developed into sub-themes. For example, the overarching theme “intrapersonal factors influencing experiences”, comprised several sub-themes such as “motor ability and perceived sport specific competence”, “sensory responses” or “emotional responses”, represented by key words and data extracts from each article, including references. For each sub-theme, both positive and challenging experiences were explored. Table 2 illustrates the procedure of thematizing the result with some examples.

**Table 2.** Example of steps in the analysis (content—codes—sub-themes—themes and overarching theme).

Overarching theme: Intrapersonal factors influencing experiences of organised physical activity			
Theme	Sub-theme	Codes	Content
Sensory responses (±)	Positive corporal and sensory experiences	*Sense of bodily fulfilment*	Feeling heavy = a good corporal feeling (Blagrave, 2017). Feeling refreshed (Obrusnikova & Cavalier, 2011). “feels good” to have muscles “working hard” (Yessick et al., 2020)
		*Sensory relief*	Getting a break from florescent light in the classroom (Blagrave, 2017).
	Negative auditory, corporal and tactile experiences	*Auditory overload*	Too noisy (Arnell et al., 2018, Haegle & Maher, 2023; Healy et al., 2013)
		*Sweating as discomfort*	Disliking getting sweaty (Blagrave, 2017; Healy et al., 2013)
Emotional responsens (±)	Experiencing joy and fun	*Enjoyment of individual activities/sports*	individual sports (Lamb et al., 2016; Obrusnikova & Cavalier, 2011), dance, gymnastics, trampolining, weightlifting (Lamb et al., 2016), boccia ball (Pellerin et al., 2022).

All articles were not represented in all sub-themes. In Table 3 in the result section all themes, sub-themes and references for included articles are presented. Finally, to assess robustness of the synthesized results, each article was scanned once again, checking the content of the article in relation to the sub-themes, making sure that the synthesis was representative for the included articles. Trustworthiness has been achieved by thoroughly working through the data and by checking the credibility of the results against this data.

**Table 3.** Thematization of factors influencing experiences of organized physical activity among children with ADHD and ASD and, experiences within these factors.

Overarching theme: Intrapersonal factors influencing experiences of organised physical activity		
Theme	Sub-theme	Content included in articles^a^
Motor ability and perceived sport specific competence (±)	Experiencing competence, skill and fitness improvement	LTPA: Arkesteyn et al. (2023); Boucher et al., (2023); Harvey et al. (2014); Ing and Mills (2019); Obrusnikova and Cavalier (2011) Arnell et al. (2018)^9^
	Lack of motor ability or fitness affecting experiences negatively	LTPA: Arkesteyn et al. (2023); Obrusnikova and Cavalier (2011); Harvey et al. (2014) PE: Arkesteyn et al. (2023); Healy et al. (2013); Arnell et al. (2018)
Sensory responses (±)	Positive corporal and sensory experiences	LTPA: Obrusnikova and Cavalier (2011) PE: Blagrave (2017); Yessick et al. (2020)
	Negative auditory and corporal experiences	LTPA: Harvey et al. (2014) PE: Blagrave (2017); Haegle & Maher, (2021); Healy et al. (2013); Yessick et al. (2020) Arnell et al. (2018)
Emotional responses (±)	Experiencing joy and fun	LTPA: Arkesteyn et al. (2023); Blagrave (2017); Boucher et al., (2023); Harvey et al. (2014); Jachyra et al. (2021), Luymes et al. (2022) Obrusnikova and Cavalier (2011) PE: Arkesteyn et al. (2023): Lamb et al. (2016); Pellerin et al. (2022). Arnell et al. (2018)
	Feeling good about oneself	LTPA: Boucher et al., (2023); Jachyra et al. (2021); Obrusnikova and Cavalier (2011) PE: Blagrave (2017); Pellerin et al. (2022) Arnell et al. (2018)
	Movement as a calming experience	LTPA: Arkensteyn, et al. (2023); Lee et al. (2014); Jachyra et al. (2021) Arnell et al. (2018)
	Feeling bored and disengaged	LTPA: Harvey et al. (2012); 2014; Luymes et al. (2022); Obrusnikova and Cavalier (2011); Jachyra et al. (2021) PE: Healy et al. (2013) Arnell et al. (2018)
	Anxiety and worrying about participation	LTPA: Arkesteyn et al. (2023); Boucher et al., (2023); Harvey et al. (2012); 2014; Jachyra et al. (2021) PE: Arnell et al. (2018); Healy et al. (2013)
	Anger and frustration triggered in organized PA	LTPA: Ing and Mills (2019),
Motivation and self- esteem (±)	Sense of meaning enhances motivation	LTPA: Arnell et al. (2018), Harvey et al. (2014) PE: Arnell et al. (2018); Arkesteyn et al. (2023); Blagrave (2017)
	Growing self-esteem through participation	PE: Blagrave (2017) Arnell et al. (2018)
	Low motivation makes participation challenging	LTPA: Arkesteyn et al. (2023); PE: Arkesteyn et al. (2023) Arnell et al. (2018)
	Self-doubts when participating in organized PA	LTPA: Ing and Mills (2019) Arnell et al. (2018)
Executive functions (-)	Challenges caused by inattentiveness	LTPA: Lee et al. (2014) PE: Haegle & Maher, (2021); Blagrave (2017)
	Challenges related to poor impulse or emotional control	LTPA: Boucher et al., (2023); Ing and Mills (2019); Lee et al. (2014)
*Interpersonal factors influencing experiences*		
Significant others (±)	Family and friends as important LTPA partners and supporters	LTPA: Arkesteyn et al. (2023); Arnell et al. (2018); Boucher et al., (2023); Harvey et al. (2014); Jachyra et al. (2021); Obrusnikova and Cavalier (2011)
Peer relations (±)	Sense of belonging	LTPA: Arkesteyn et al. (2023); Harvey et al. (2014); Ing and Mills (2019); Lee et al. (2014); Obrusnikova and Cavalier (2011) E: Haegele and Maher (2022); Healy et al. (2013); Pellerin et al. (2022); Yessick et al. (2020).
	Not feeling at ease in the group	PLTPA: Arkesteyn et al. (2023); Lee et al. (2014) PE: Haegele and Maher (2022) Arnell et al. (2018)
	Being subjected to bullying	LTPA: Jachyra et al. (2021) PE: Haegele and Maher (2022); Healy et al. (2013)
	Negative social comparisons with peers	PE: Arnell et al. (2018); Healy et al. (2013)
Coach/teacher relations (±)	Appreciation of a caring and responsive coach/teacher	LTPA: Arkesteyn et al. (2023); Ing and Mills (2019); Lee et al. (2014) PE: Arkesteyn et al. (2023); Arnell et al., (2018); Blagrave (2017); Boucher et al., (2023); Yessick et al. (2020)
	Challenges in interactions between coaches and athletes/teachers and students	LTPA: Arkesteyn et al. (2023); Jachyra et al. (2021); Lee et al. (2014) PE: Blagrave (2017)
*Institutional factors influencing experiences*		
The level of structure, predictability and adaption offered by the institution (±)	Feeling safe through routines and familiar settings	PE: Arkesteyn et al. (2023); Yessick et al. (2020). Arnell et al. (2018)
	Lack of adaptability in PE	PE: Arnell et al. (2018); Healy et al. (2013)
*Community and policy factors influencing experiences*		
Opportunity and availability	Opportunity and availability facilitate LTPA	LTPA: Arkesteyn et al. (2023); Obrusnikova and Cavalier (2011)
	Lack of opportunity and availability hindering LTPA	LTPA: Arnell et al. (2018); Boucher et al., (2023); Harvey et al. (2014); Obrusnikova and Cavalier (2011)
Hindering policies and regulations	Rules and regulations perceived as having a negative influence	LTPA: Jachyra et al. (2021) PE: Arkesteyn et al. (2023)
*Physical factors influencing experiences*		
Outdoor environment	Positive to be outside/in nature	Arkesteyn et al. (2023)
	Impact of weather and temperature on participation and negative experience	LTPA: Arkesteyn et al. (2023); Obrusnikova and Cavalier (2011) PE: Arkesteyn et al. (2023) Arnell et al. (2018)
	Bugs affected participation and experiences negative	LTPA: Obrusnikova and Cavalier (2011) Arnell et al. (2018)
Indoor environment	Adequate space and a pleasant gym environment	PE: Arkesteyn et al. (2023); Blagrave (2017); Lamb et al. (2016)
	Lack of space	LTPA: Harvey et al. (2014) PE: Arkesteyn et al. (2023)
	Too much noise make experience unpleasant	LTPA: Harvey et al. (2014) PE: Arkesteyn et al. (2023)
	Changing rooms as a chaotic and unpleasant environment	PE: Arnell et al. (2018); Lamb et al. (2016)
Equipment	Lack of safe equipment	LTPA: Arkesteyn et al. (2023); Obrusnikova and Cavalier (2011); Harvey et al. (2014) PE: Healy et al. (2013)

^a^Harvey et al., 2012 and 2014; Ing and Mills, 2017; Lee et al., 2014; Luymes et al., 2022.

## Result

### Overview of previous research

The findings of the literature review demonstrate that research within this field has been conducted across various countries, although there is an overweight towards the USA (*n* = 6) and Canada (*n* = 6). Most studies were carried out with children diagnosed with ASD (*n* = 12), whereas fewer studies have explored experiences among children diagnosed with ADHD (*n* = 4), or comorbidity of the two (*n* = 1). All five studies in which children with ADHD were included are within the context of LTPA. Further, studies typically involve adolescents, with some also encompassing younger children. Ten studies include both girls and boys; however, it is notable that none of these include many girls. Six studies contain only boys, and one is a case study of one girl and no boys. Interviews are the most used method, and several studies have included prompts such as photos or pictures to improve communication. Table 1 describes the characteristics of each included study. Organized physical activity has been divided into leisure time physical activity (LTPA), including organized physical activities preformed during spare time and, PE for activities preformed during lessons in school.^6^

### Experiences of organised physical activity of children with ADHD and ASD

The result of the synthesis describes physical activity experiences among children diagnosed with ADHD and ASD. The result presents each overarching theme derived from the socio-ecological model, encompassed by various themes focusing on factors influencing experiences and sub-themes that describe children’s experiences within each factor. In each theme, both positive and negative aspects are highlighted (see Table 3).

### Intrapersonal factors influencing experiences

The meta-synthesis demonstrates that ADHD and ASD-diagnosed children have mixed experiences—both positive and negative—of organized physical activities, including community-based physical activity programmes, PE, adapted PE, self-contained PE, sports and adapted sports.

### Motor ability and perceived sport-specific competence

The children in Arnell et al. (2018)’s study stated that in order to be able to participate in physical activity they needed a certain minimum level of physical competence. If they perceived that they did not have this, they would probably not participate. Research conducted on children with ADHD/ASD have shown that they display more motor ability difficulties than neurotypical peers (Green et al., 2009; Harvey et al., 2009, 2014).The sub-theme *lack of motor ability or fitness affecting experiences negatively* includes challenges and bad experiences from participation in organized physical activity (Arkesteyn et al., 2023; Arnell et al., 2018; Harvey et al., 2014; Healy et al., 2013; Obrusnikova & Cavalier, 2011). Such as “Glen” in the study by Harvey et al. (2014, p. 215) saying: “Yeah but I don´t like it (racket sports) because I don´t have a lot of precisson, you know”.

The literature review also demonstrates that participants have positive experiences. Children reported that they *experience competence, skill and fitness improvement* when participating in organized physical activity. These positive experiences consist of feelings of competency, shown among ASD-diagnosed children in certain types of physical activities, particularly those to which they were accustomed to and considered to be uncomplicated (Arnell et al., 2018). Further, adolescents with ADHD had specific knowledge of the movements required and perceive that they had the necessary skills for playing sports (Harvey et al., 2012, 2014). This sub-theme also show that some children experience that they are gaining motor ability and fitness through participation in physical activity (Arkesteyn et al., 2023) and that participation in PE/APE developed skills necessary for doing physical activity and exercise (Obrusnikova & Cavalier; Blagrave, 2017).

### Sensory responses

Sports and physical activity generate various physical stimuli (see further physical factors) that can elicit sensory responses in participants. Research indicates that individuals with neurodevelopmental disorders, including ADHD and ASD, exhibit heightened sensitivity to sensory stimuli, potentially rendering the environments in which sports and physical activities are conducted particularly challenging. In the reviewed articles, negative responses to stimulus were foremost *auditory and corporal*. Several studies pointed to an overload of sound during physical activity (Arnell et al., 2018; Haegele & Maher, 2022; Healy et al., 2013; Yessick et al., 2020). Other reactions were corporal, such as disliking the feeling of sweat (Blagrave, 2017; Healy et al., 2013). In a few cases, organized physical activity was described as containing *positive corporal and sensory experiences* such as “feeling heavy” (Blagrave, 2017) or feeling refreshed by taking part in physical activity (Obrusnikova & Cavalier, 2011) as positive feelings. Or as “Andy” explains “it ‘feels good’ to have his muscles ‘working hard’ while doing the activity” (Yessick et al., 2020, p. 56). In this sense organized physical activity may contribute to a sense of bodily fulfilment.

### Emotional responses

Organized physical activity can evoke a wide range of emotions, both positive and negative. A sub-theme presenting positive emotions among children is the *experience of joy and fun* in organized physical activity. Both individual and team activities were found to be enjoyable. Specific individual activities mentioned as positive included gymnastics, trampolining, weightlifting, bowling, bocce ball, and dance (Lamb et al., 2016; Obrusnikova & Cavalier, 2011; Pellerin et al., 2022). Playing games and team sports were also described favourably in some studies, including sports such as baseball and basketball (Boucher et al., 2023; Lamb et al., 2016; Obrusnikova & Cavalier, 2011; Pellerin et al., 2022). However, children diagnosed with ASD mostly referred to small-sided games, activities with limited and explicit rules, minimal requirements for language interpretation, or restricted aspects of team games—such as shooting a ball at a hoop (Lamb et al., 2016). Furthermore, Arnell et al. (2018) illustrate that play was preferred over more serious team games. Competitive elements were appreciated by some children but experienced as negative by others (Arkesteyn et al., 2023; Arnell et al., 2018). An essential component of enjoyment involved successful engagement in activities (Blagrave, 2017; Pellerin et al., 2022).

Further, positive emotional responses to organized physical activity are captured in the sub-theme *feeling good about oneself* in organized PA (Jachyra et al., 2021), such as participation in LTPA contributing to a “good feeling” (Boucher et al., 2023) and experiencing a sense of wellbeing (Arnell et al., 2018; Blagrave, 2017). Another sub-theme describes *movement as a calming experience*. Physical activity helped burn excessive energy (Lee et al., 2014), was described as an emotional venting or outlet (Jachyra et al., 2021; Lee et al., 2014), or a respite from stress (Arnell et al., 2018). These examples illustrate the significant role that physical activity can play in the positive emotional experiences among children and adolescents with ADHD and ASD.

Unfortunately, children also associated several negative feelings with organized physical activities, explained in the sub-theme *feeling bored and disengaged*. Harvey et al. (2012, 2014) found that some children diagnosed with ADHD showed a dislike of leisure time physical activities, including experiences of finding physical activity too demanding. Similar results were found among children diagnosed with ASD (Arnell, et al., 2018; Healy et al., 2013). Further, activities were perceived as uninteresting (Jachyra et al., 2021; Obrusnikova & Cavalier, 2011). Some specific elements were also found to be boring, such as dance (Arnell et al., 2018; Luymes et al., 2022) and, for some, competitive activities (Arnell et al., 2018).

Physical activity also generated *anxiety and worrying about participation* (Arkesteyn et al., 2023; Arnell et al., 2018; Jachyra et al., 2021), and in addition, more specific types of anxiety were found, such as performance anxiety (Harvey et al., 2014), body appearance-anxiety (Arnell et al., 2018), or fear of getting hurt (Boucher et al., 2023; Healy et al., 2013). Participation in organized physical activities sometimes led to *anger and frustration*, directed both at others and at the children themselves (Ing & Mills, 2019).

### Motivation and self-esteem

Motivation is a crucial facilitator for engaging in sports (Obrusnikova & Cavalier, 2011). Physical activity became motivating for the participating children when they perceived a *sense of meaning* by participating in activities. This was experienced when activity was perceived as important—and when they were associated with a goal or health benefits, such as gaining health (Harvey et al., 2014) or mental health (Blagrave, 2017) or doing physical activity with an intention to to stay fit (Arkesteyn et al., 2023). Also, fear of ill-health or obesity served as a motivator for engaging in health-enhancing activities (Arnell et al., 2018). Other aspects that brought meaning to the activity was to achieve results in sports (Arkesteyn et al., 2023). Further, autonomy and freedom of choice enhanced motivation to participate in activities (Arkesteyn et al., 2023; Arnell et al., 2018). Additionally, participation in organized physical activity were experienced as helping to *grow self-esteem* (Arnell et al., 2018; Blagrave, 2017).

Further, the synthesis show that *low motivation makes participation challenging* (Arkesteyn et al., 2023; Arnell et al., 2018). Both studies identified a lack of motivation to engage in physical activity among children with ASD. Even when activities were deemed enjoyable, some children in the study by Arnell et al. (2018) found it difficult to initiate physical activity. Moreover, experiences of *self-doubt when participating* in organized PA, was found among children with ADHD (Ing & Mills, 2019) and a lack of confidence, feelings of insecurity, and low self-esteem was found among children with ASD (Arnell et al., 2018).

### Challenges in executive functions

ADHD and ASD are associated with disability-specific constraints, some of these are challenges in executive functions. In the reviewed research articles, some of these constraints are described in relation to their contributions to negative experiences of organized physical activity. First, *challenges caused by inattentiveness* in organized physical activity, were described as “drifting off” and caused difficulties in following instructions (Haegele & Maher, 2023; Lee et al., 2014).

*Lack of control* caused difficulties especially among children with ADHD, which is described in studies by Ing and Mills (2019) and Lee et al. (2014). Impulsive behaviour, such as blurting out comments (Lee et al., 2014) to other children or deliberately tackling someone in a ball game (Ing & Mills, 2019), caused difficulties for some children. Lee et al. (2014) gives an example of this with a statement from “Michael”: “I blurted out a comment. I said something about him like just getting stupider and stupider. And I stopped and I was like ‘Why would I say that’?” (p. 350). Children diagnosed with ASD also portrayed how lack of control, such as biting or hitting other participants prevented them from playing sports (Boucher et al., 2023).

## Interpersonal factors influencing experiences

Friends and family, as well as teammates and coaches are important in shaping both negative and positive experiences of organized physical activity.

### Significant others

Significant other’s such as family and friends, play a crucial role in influencing participation in, and experiences of, physical activity. The sub-theme *family and friends as important LTPA partners and supporters* indicates the necessity of having significant others facilitate engagement in PA during leisure time (Arnell et al., 2018; Blagrave, 2017; Boucher et al., 2023; Harvey et al., 2014; Jachyra et al., 2021; Obrusnikova & Cavalier, 2011). Conversely, lacking companions to participate with or lack of parental support can hinder participation in LTPA (Arkesteyn et al., 2023; Obrusnikova & Cavalier, 2011).

### Peer relations

According to Healy et al. (2013, p. 41), PE “can socially benefit children with ASD”. Positive experiences seem to occur when the child feels accepted (Ing & Mills, 2019) and experiences friendship in the team or group (Arkesteyn et al., 2023; Healy et al., 2013; Luymes et al., 2022; Yessick et al., 2020). This is captured in the sub-theme *sense of belonging* in organized PA and is exemplified by Haegele and Maher (2022)’s study in which one student (“Chris”) noted developing strong relationships with his classmates in PE, which he attributed to his ability as an athlete and a shared sense of group athleticism. Further, sharing a common goal (Lee et al., 2014) and working as a team (Harvey et al., 2014), are shown to be important to develop and keep friendship going. In a study of self-contained PE by Pellerin et al. (2022), a sense of belonging was identified as the importance of gaining friendships with other children with disabilities.

In contrast, the sub-theme *not feeling at ease in the group* captures challenges in peer relations, such as the feeling of being in an unpleasant group (Arkesteyn et al., 2023) or experiencing difficulties communicating to, or adjusting to other children (Arkesteyn et al., 2023; Arnell et al., 2018). This may lead children to reduced involvement in team sport or group activities (Arnell et al., 2018). Haegele and Maher (2022) identify that a barrier to forming friendships in PE is the lack of shared interests, i.e., where autistic students found themselves having different interests compared to their peers. In the most severe cases of negative peer relations, children were *subjected to bullying* from other children, primarily through verbal means, but in some cases, physically (Haegele & Maher, 2022; Healy et al., 2013; Jachyra et al., 2021). Another form of bullying is exclusion by other children, such as singling out the child with a disability (eg. being selected last for a group or not being included in play or games). This also involved situations where the children were perceived as lacking ability, and other children verbally accused them for not being able to play the game correctly, for example, as “Bill” expressed: “They keep saying that ‘Bill can’t catch a ball’” (Healy et al., 2013, p. 225).

Another issue concerns the impact of negative social comparisons with peers, described in PE. Such comparisons may relate to performance, or to perceiving other children’s competitiveness as distressing. Some children with ASD described feeling less energetic than their peers and reported feeling bad when they came last in competitions (Healy et al., 2013). Other social comparisons were related to physical appearance, such as low perceived body satisfaction, particularly during situations like changing clothes or showering in PE (Arnell et al., 2018). Furthermore, children in Arnell et al’s study described feeling uneasy when under scrutiny or evaluation by others, even when the assessment was positive.

### Coach/Teacher relations

Results of the literature review shows that *appreciation of a caring and responsive coach/teacher* was important to experience organized physical activity in a positive manner (Arkesteyn et al., 2023, Arnell et al., 2018; Blagrave, 2017, Boucher et al., 2023; Ing & Mills, 2019; Lee et al., 2014; Yessick et al., 2020). Among children included in the studies, several had positive experiences of their PE/adapted- och self-contained PE teacher (Arkesteyn et al., 2023, Arnell et al., 2018; Blagrave, 2017, Boucher et al., 2023; Yessick et al., 2020). As an example, Blagrave (2017) cites children feeling that the PE teacher was helpful and friendly. An important aspect highlighted by the children was to have a teacher that was “keen, responsive and clear, besides being understanding of their abilities and needs” (Arnell et al., 2018, 1798). Similar results are found in after-school activities such as sports. Research by Lee et al. (2014) indicate that children with ADHD who felt that coaches were supportive and worked patiently with them made them feel positive about taking part in sport. The importance of a caring and understanding coach is also evident in the retrospective story by Ing and Mills (2019). The authors stress the need of coaches to “remain patient and develop the trust and respect required to make athletes feel competent and supported” (p. 7).

Unfortunately, not all relationships were experienced as positive. Challenges in *interactions between coaches and athletes, or teachers and students*, were primarily addressed in LTPA (Arkesteyn et al., 2023; Jachyra et al., 2021; Lee et al., 2014). A study by Lee et al. (2014) found that children with ADHD perceived emotional reactions from coaches, such as frustration or anger, negatively. For instance, athletes reported adverse experiences when coaches responded negatively to their mistakes. Another challenge relates to children’s distractibility (cf. *executive functions*), and the redirection they receive from teachers or coaches, which has been reported to result in negative experiences (Lee et al., 2014; cf.; Blagrave, 2017, for APE). Children also encountered difficulties when coaches failed to adequately consider their needs. For instance, Jachyra et al. (2021) describe how excessive verbal instructions from coaches can make it challenging for children who have greater difficulties with many instructions. There are also cases of exclusion from teachers or leaders in sports. In PE, examples are show to how the PE teacher directs the child to do something else while for example playing a ball game. In sports, a child may be sent away from the playing field due to incidents occurring such as tackling another player unfairly—this is experienced as exclusion from the child’s point of view (Ing & Mills, 2019).

## Institutional factors influencing experiences

Physical activities are organized in different arenas such as schools, sports clubs, and community based physical activity centres, each which may offer unique experiences. Few articles described experiences of institutional factors, those that did are captured in the theme *Levels of structure, predictability and adaption offered by the institution*. The positive aspects of this theme are exemplified in Arkesteyn et al. (2023), where children express that participating in LTPA held at the same location and at the same time was beneficial for their engagement. Similarly, performing familiar activities in a familiar setting was described as a positive experience according to Arnell et al. (2018) and having consistent routines and visual targets and trackers was experienced as positive in PE (Yessick et al., 2020). In essence, physical activity preformed in an institution providing safety through routines and familiar settings are experienced positive. In contrast, a lack of predictability was regarded as negative (Arnell et al., 2018).

In two articles, PE was specifically considered to be an institution with *lack of adaptability*, by being perceived as both unpredictable and inflexible. Some children found lack of adaptions to their needs in PE, which in turn limited their participation (Arnell et al., 2018). Further, according to children diagnosed with ASD, PE was experienced as negative due to rigidity, restricting freedom of choice in activities and, lack of influence over who to do an activity with (Arnell et al., 2018). Lack of adaptability in PE, may lead to self-exclusion by the child. In some cases, children choose to self-exclude by not wanting to participate in some activities in PE (Healy et al., 2013) or to request removal of PE classes from their individual education plan (Arnell et al., 2018).

## Community and Public-policy factors influencing experiences

Few of the included studies provide information on how children perceive community and public-policy factors as influencing their experiences of organized physical activity. When such factors are mentioned by children, they tend to relate to experiences of *opportunity and availability* to participate in organized activities. Access to appropriate leisure-time LTPA was described as positive and seen as a facilitator of participation (Arkesteyn et al., 2023; Obrusnikova & Cavalier, 2011). However, several children reported a lack of opportunity and availability, including barriers such as a shortage of adapted sport activities (Boucher et al., 2023), activities being too time-consuming or costly (Harvey et al., 2014), and a lack of transportation to and from suitable activities (Arnell et al., 2018; Obrusnikova & Cavalier, 2011).

*Hindering policies or regulations* may act as a barrier to participating in organized physical activity or negatively influence the experience. Jachyra et al. (2021) demonstrate exclusion from activities of interest due to service providers requiring a personal support worker for participation. In cases where this support was unavailable, children were unable to join these activities. In a study by Arkesteyn et al. (2023) some children considered the mandatory aspect of PE as negative. Furthermore, dressing rules and rules concerning transportation to and back from the PE gym were experienced as negative.

### Physical factors influencing experiences

#### The outdoor environment

*Weather and temperature* had an *impact on participation and shaped negative experience* of physical activity in the outdoor environment. Temperature (e.g., too hot or too cold) as well as weather (e.g., wind, rain or snow) had a negative and limiting effect, or excluded participation altogether (Arkesteyn et al., 2023; Arnell et al., 2018; Obrusnikova & Cavalier, 2011). Similar results were found in a questionnaire by Stanish et al. (2015) showing that 81% of children diagnosed with ASD reported that, in their opinion, it was sometimes too hot or cold to do physical activity and most of these reported that this hindered them from partaking. Likewise, *bugs* in the outdoor surroundings may hinder children’s participation or negatively affect their experience (Arnell et al., 2018; Obrusnikova & Cavalier, 2011). Only one study acknowledges positive experiences of the outdoor context, in which being outside and expiring nature was highlighted as positive (Arkensteyn et al., 2023).

#### The indoor environment

*Adequate space and a pleasant gym environment* to do physical activity in, is highlighted in some of the studies. On the positive side, some children in a study by Arkesteyn et al. (2023) talked about experiencing a spacious and quiet gym and that this facilitated their participation in PE. The PE gym was also described as a relief from other negative sensory stimulus such as bright light in the classroom in a study by Blagrave (2017). Children in Lamb et al. (2016) study made positive associations with the PE teacher’s office when talking about spaces for PE. Apart from these studies, most studies acknowledge the indoor environment as less pleasant.

Physical aspects like *too much noise make experience unpleasant*. The physical activity context is generally experienced as loud or noisy (Arkesteyn, 2023; Arnell et al., 2018; Harvey et al., 2014). For instance, a boy in Arnell et al. (2018)’s study describes dancing as a loud activity that made him tired and unable to focus. Or as “Greg” a child with ADHD perceived sports participation: “Sometimes there are parents that are screaming and it´s hurting my ears” (Harvey et al., 2014, p. 215).

In Arnell et al. (2018) and Lamb et al. (2016) articles, *changing rooms* were specifically described as a *chaotic and unpleasant area*, evoking negative feelings associated with participating in PE. The corridor leading up to the PE gym was also experienced as crowded and noisy (Lamb et al., 2016). Further, Haegele and Maher (2022) emphasize the changing room as an especially problematic physical space due to occurrence of bullying.

Lastly, as children with ADHD and ASD appear to have an increased fear of getting hurt, the feeling of unsafety in the environment was sometime driven by physical factors such as equipment perceived as dangerous (Arkesteyn et al., 2023; Harvey et al., 2014; Healy et al., 2013; Obrusnikova & Cavalier, 2011).

## Discussion of synthesized research

The research overview presented in Table 1, demonstrates that there is an overall lack of research into experiences of organized physical activity among the studied population, from children’s own perspective and, few of the studies included children diagnosed with ADHD. Further, girls are frequently underrepresented both in studies on ADHD and ASD. It can thus be stated that we specifically need more knowledge about experiences among ADHD diagnosed children and further, advance knowledge on ADHD/ASD diagnosed girls’ experiences of organized physical activity.

Research is produced in several countries, with a greater proportion of the research is in USA and Canada whereas there is a lack of studies in South America, Asia and Africa. Further, studies in the Nordic countries are scarce, with only one Swedish study included.

The synthesis demonstrates that children have both positive and negative experience of organized physical activity. These experiences are shaped by intrapersonal factors, such as their individual needs and abilities, interpersonal factors, including their relationships with others, and various external factors at the institutional, community or policy level. The latter often pertains to the availability and adaptation of activities, with institutional factors specifically relating to the extent of accommodations made for individual needs. Interestingly, PE was portrayed in some studies as an institution lacking adaptability, while in others it was depicted as offering structure and predictability. This suggests the need for further studies on how institutionalized aspects of PE affect experiences. Given that intrapersonal and interpersonal factors are more immediate to the child and frequently cited in descriptions of their experiences, the discussion will primarily focus on these factors. The results will further be compared with other relevant studies, and implications from the literature review will be discussed.

In understanding the research, it is important to consider how experiences of physical activity may be impacted by the nature of ADHD and ASD, and how these shape the lives and reactions of those with those conditions. For instance, certain intrapersonal factors that negatively influence experiences of organized physical activity may be considered disability-specific, such as challenges related to executive functioning. Inattention was challenging, especially in relation to following instructions. Impulsivity causes difficulties for some when interacting with others and may in turn cause challenges in peer relations due to the child not acting as expected by others (eg. blurting out comments). These results are consistent with parental reports suggesting that disability-specific constraints may pose additional challenges for children with ADHD or ASD (Ayvazoglu et al., 2015; Blagrave & Colombo-Dougovito, 2019; Gürkan & Kocak, 2020; McMahon et al., 2020). Further, as explained at the beginning of the article, research show that motor difficulties are more common among children with neuro developmental disorders (Green et al., 2009; Harvey et al., 2014). In the analyzed articles, lack of motor ability or fitness caused difficulties in participating in activities and, for some, generated feelings of inadequacy. Another negative experience was related to sensory responses, with difficulties in tolerating sweat, noise or bright light. High sensitivity to stimuli is common in children with ASD (Yessick et al., 2020). The synthesis aligns with other research suggesting that the disability itself may pose additional challenges in participation in activities (compare Arnell et al., 2020; Nichols et al., 2019). Based on these results, it is important that coaches/teachers are aware of the challenges that may be specific to children with ADHD and ASD and potential strategies and solutions, to enable them to adjust their approach to reflect children’s needs.

Further, participation in physical activity provoked negative feelings of boredom, stress and anxiety among some of the children, as well as low self-esteem and lack of motivation (cf. Hickingbotham et al., 2021). This implies that coaches/teachers need to ensure physical and mental support for children. Drawing on research by Jones and Thomas (2015), and the term “pedagogical scaffolding”, I suggest that scaffolding could help children with ADHD/ASD to feel safe, and motivated and develop self-confidence while learning through sports with the support of the coach. I base this suggestion on the view of scaffolding as a mediator in the learning process taking into consideration both the context in which learning is taking place (macro and meso level) and the actual interaction within this context (micro level).

On the positive side, several children enjoyed physical activity and experienced joy as a result of their participation. Although many children perceive motor difficulties as a hindrance, the literature review indicates that organized physical activity can enhance motor skills and boost confidence, enabling participation in other activities. Furthermore, some studies suggest that such activities can improve self-esteem and highlight the importance of motivation for children’s participation. These findings align with parental reports suggesting that sports activities contribute to development in various life aspects, both within and beyond the realm of sports (Gürkan & Kocak, 2020; Kristén et al., 2023; May et al., 2018; Rodriquez et al., 2022). Based on the review, it is important to include and to keep children with ADHD and ASD in organized activities so that it can help them improve their abilities and confidence well enough to promote participation in sports and PE.

The synthesis further highlights how interpersonal relations may cause difficulties for ADHD and ASD-diagnosed children. Results demonstrate negative experiences due to a lack of adaption among coaches and teachers. Also, when coaches or teachers reacted emotionally or in frustration at children’s behaviour, children found it difficult to bear. This result is in line with research on parent’s perceptions about coaches, which highlights the challenges of lack of adaption and greater use of intimidating coaching among coaches working with hyper-active children (Vargas et al., 2019). Further, other research demonstrates that coaches lack experience, knowledge and practice in training children with neuro developmental disabilities (Ayvazoglu et al., 2015; Blagrave & Colombo-Dougovito, 2019; Gregor et al., 2018; Gürkan & Kocak, 2020; Vargas et al., 2015). On the positive side, some of the children in the research reports talked positively about their coaches and teachers and found them kind and caring. A supportive coach-athlete/teacher-student relationship was indicated as important for children with ADHD and ASD. Similar results are shown in a study of children with physical disability in the school subject PE. The importance of a strong relationship between PE teacher and child was demonstrated as a positive factor to participate (Fitzgerald, 2005).

Additionally, some research indicates that children experienced exclusion and even bullying from their peers. This aligns with findings on leisure activities (Brewster & Coleyshaw, 2011) and PE experiences among children with other disabilities or special educational needs (Coates & Vickerman, 2008; Fitzgerald, 2005; Fitzgerald & Stride, 2012; Haegele & Sutherland, 2015). Further, social comparison was perceived negatively by the participating children, similar to findings in a study on children with disabilities in PE (Fitzgerald, 2005). However, not all interpersonal experiences are negative. Although previous research has shown that children with ASD are less inclined than typically developed children to view organized physical activity as a way to make friends (Stanish et al., 2015), this literature review indicates that sports activities or (adapted) physical education in school can facilitate friendships for individuals with ADHD and ASD (Harvey et al., 2014; Healy et al., 2013; Lee et al., 2014). Similar results were found in an earlier study by Harvey et al. (2009) and in parental reports (Gürkan & Kocak, 2020). Based on these findings, activities must encourage cooperation and inclusiveness to help children form friendships through organized physical activity.

A limitation of this study is the difficulty in identifying qualitative research that provides a firsthand perspective from children diagnosed with ADHD and ASD. Several records did not meet the inclusion criteria, which raises the possibility that relevant studies may have been overlooked due to the search terms used. It is conceivable that alternative search terms could have resulted in the inclusion of additional articles. However, it is important to note that multiple search terms were tested prior to finalizing the selected terms, and these did not yield any records beyond those identified through the chosen terms. Furthermore, a librarian was consulted to ensure the most effective search strategy was employed.

Furthermore, the two overarching themes most frequently addressed in the included research articles are intra- and interpersonal factors influencing experiences of organized physical activity. Within these themes, all sub-themes (though not all individual codes) included research involving both study populations. However, it should be noted that research focusing on the experiences of children with ADHD remains limited, and firm conclusions cannot be drawn. Moreover, none of the overarching themes related to institutional factors or community and policy factors influencing experiences were represented in studies involving children diagnosed with ADHD. This may reflect a lack of research attention to these areas. Furthermore, although ADHD and ASD are often comorbid, they are heterogeneous conditions. Even within each diagnosis, children may have diverse perceptions of their condition and how it affects them (Ringer, 2020). Efforts were made to present the results in a way that clearly indicates whether findings apply to one or both diagnoses. Further, it should be acknowledged that some studies have limited inclusion to children with an IQ over 70 and/or sufficient verbal abilities to engage in interviews. While this enhances the credibility of the findings, as participants are better able to articulate their experiences, it also narrows the total scope of the findings. The exclusion of children with more significant cognitive or communicative challenges means that important perspectives are missing, which limits the transferability and richness of the insights. This highlights the need for further research that includes children with a wider range of communicative and cognitive profiles, to develop a more inclusive understanding of their experiences.

The term “organized physical activity”, further encompasses various forms, including PE in schools (both adapted, self-contained and integrated), community-based physical activity centres, and sports. Despite this diversity, research indicates that many experiences are similar across these different activities. When I have found it important, I have specified the context from which each result originates to ensure clarity. The context of PE and LTPA is also indicated in Table 3 to ensure that the reader can identify which type of activity each theme or sub-theme relates to. Notably, none of the studies focusing on children diagnosed with ADHD included experiences of the school subject PE.

A further limitation is the high representation of reports from the USA and Canada which may affect the generalizability of the overall result. Readers should keep in mind that physical activity contexts may be different in other countries. One last limitation is that a qualitative meta-synthesis although helping to get a fuller picture of a phenomenon also includes a reinterpretation of research. As such, the results presented are “two stages away from the individual’s own words” (Ringer, 2020, p.), meaning that there is a risk of losing part of the experiences expressed by the participating children. To limit this risk, I have given several examples from the original articles to strengthen the interpretations made, while at the same time, keeping close to the research that the synthesis builds upon. I have also, when appropriate, included some original quotes to provide examples of and emphasize the children’s voices.

## Conclusion and further implementations

To conclude, the synthesis of qualitative studies highlights the diversity of experiences among individual children with ADHD and ASD about organized physical activity. The results highlight specific aspects that can inform further research and efforts to include children with ADHD and ASD in various forms of organized physical activities.

Although several studies were found in total on physical activity and children diagnosed with ADHD or ASD, only a few explored children’s own *experiences*. There is a particular lack of studies including children with ADHD and girls with both ADHD and ASD. As a researcher at a Swedish university, I recognize the need for more research within a Swedish/Nordic context. Overall, there is a need for more research that incorporates first-hand perspectives from the children themselves.

Since ADHD and ASD are common disabilities among children (with ADHD affecting 5–8% and ASD 1%), it is crucial for their physical and mental health and well-being that they do not miss out on the benefits of organized physical activity. This highlights the need for teachers, leaders, and coaches to acquire more knowledge on how to support these children. Understanding what matters to them can help inform institutions such as schools and sports organizations on how to empower children in and through organized physical activities. The findings clearly show that children have valuable insights that stakeholders in organized physical activities must consider. It is essential that this information is heard and acted upon to create inclusive and supportive environments for all children.

## Supplementary Material

Manuscript_Grahn_Karin_revision2_250609.docx

Appendix 1 ENTREQ Checklist.docx

## Data Availability

All included articles are published online.

## Notes

1. 5–8% of children are diagnosed with ADHD (World Health Organisation, WHO, 2019) and about 1% have an ASD diagnosis (Zeidan et al., 2022). Boys are more often diagnosed than girls.

2. Children are all humans up to the age of 18, according to the United Nation’s definition; however, when a report specifically includes adolescents (ages 10–19 according to the World Health Organisation’s definition) this term will also be used.

3. Adapted forms of PE and sports are adjusted to the person with a disability. Adapted forms of sport/PE can take place in a general PE class/sport team with accommodations or, in small groups, or in an individual setting, depending on the needs of the child. Self-contained physical education is an alternative class specifically designed for students with disabilities who risk not to learn satisfactory within integrated physical education. Activities are usually preformed in smaller groups and with specific adaptions.

4. Search for literature published 2010–2021: SPORT DISCUS (13–02–22), ERIC (15–02–22), EDUCATION DATABASE (15–02–22), SCOPUS (16–02–22), WEB OF SCIENCE (sport science och Education special, 16–02–22), GU search (11–03–22). Search for literature published 2022–2023: SPORT DISCUS (12–04–24), ERIC (12–04–24), EDUCATION DATABASE (12–04–24), SCOPUS (12–04–24), WEB OF SCIENCE (sport science och Education special, 06–05–21), GU search (07–06–24). Reference lists included in the sample have been scanned for additional references.

5. Neuro developmental disorders such as ADHD and ASD are sometime referred to as “hidden disabilities” since they are disabilities that do not show from an outside perspective.

6. For the clarity of this literature review, I have used the concepts LTPA and PE to categorize activities in Table 1. The categorization is based on descriptions of activities in the included articles. In case an article contain result on other activities that cannot be categorized as organized physical activity (i.e., playing computer games or waking the dog) these results have not been included in the synthesis.

7. Organized physical activity is categorized as LTPA and PE. If specified I have also added the type of LTPA and PE that are included, such as sport, APE etc. For clarity, I have only included physical activities included in the analysis. In case the report also includes unorganized PA or other leisure time activities that do not include physical activity they are not included in the table.

8. Comorbid diagnosis including oppositional defiant disorder (ODD), conduct disorder (CD) and separation anxiety (SA).

9. Arnell et al. (2018), Arkesteyn et al. (2023), and Boucher et al. (2023) use the broader concept of physical activity (PA), which encompasses both leisure-time physical activity (LTPA), such as organized sports, and the school subject of physical education (PE). When it was possible to clearly determine whether the findings related specifically to either PE or LTPA, the references were categorized accordingly. However, when the term PA was used in a more general sense and such distinctions could not be made, the references were placed at the end of the list under each sub-theme.
