# Supplementary material for: Physical activity experiences among children with ADHD and ASD: a qualitative meta-synthesis literature review
Source: Int J Qual Stud Health Well-being. 2025 Jul 23;20(1):2524460. doi: 10.1080/17482631.2025.2524460 (PMC12291193; doi:10.1080/17482631.2025.2524460)
Supplement: Manuscript_Grahn_Karin_revision2_250609.docx [file ZQHW_A_2524460_SM2620.docx]

# Physical activity experiences among children with ADHD and ASD: A Qualitative Meta-Synthesis Literature Review

Karin Grahn

Department of Food and Nutrition and Sport Science, University of Gothenburg

https://orcid.org/0000-0002-0166-7582

# Physical activity experiences among children with ADHD and ASD: A Qualitative Meta-Synthesis Literature Review

Karin Grahn, Department of Food and Nutrition and Sport Science

https://orcid.org/0000-0002-0166-7582

## Abstract

**Purpose:** The aim was to analyse and synthesise empirical research on physical activity experiences among children diagnosed with ADHD and ASD.

**Methods:** A qualitative meta-synthesis was conducted, including 17 articles published between 2010 and 2023. The synthesis encompasses qualitative research on the experiences of children with ADHD and ASD in organised physical activity. Data were categorised into overarching themes based on a socio-ecological model. Each overarching theme was subdivided into themes and sub-themes illustrated with extracts from each study.

**Results:** Research was conducted in several countries, with a predominance of studies from USA and Canada. Most studies focused on children diagnosed with ASD, with boys being more frequently represented. Various intrapersonal factors were influential, with negative factors including disability-specific constraints, motor skill difficulties, and dissatisfaction with physical activity, while enjoyment of specific activities and positive attitudes towards physical activity were identified as some of the positive factors. In terms of interpersonal factors, interactions with family, friends, teammates, and coaches or teachers shaped both positive and negative experiences.

**Conclusions:** The findings provide valuable insights into the experiences of children with ADHD and ASD in organised physical activity contexts. Key aspects identified can guide future research and initiatives aimed at including children with ADHD and ASD in organised physical activities.

**Key words:** disability, attention deficit hyperactivity disorder, autism spectrum disorder, sport, physical education, socio ecological model, qualitative research

**Word count:** 12 502 words (including tables and figures).

## Introduction

Attention deficit hyperactivity disorder (ADHD) and autism spectrum disorder (ASD) are neurodevelopmental disabilities leading to impairments influencing everyday life^[[1]](#footnote-1)^. ADHD affect children’s attention, impulse-control, and activity levels; while ASD consists of impairment in social interaction, communication, and behavioural rigidity (American Psychiatric Association, 2013). ADHD and ASD have also been shown to interrelate with poor movement skills (Green et al., 2009; Harvey et al., 2014). One manifestation of these conditions is their impact on their engagement with and experience of physical activity. Children diagnosed with ADHD and ASD are generally less physically active, less likely to meet the recommended physical activity levels and have more negative experiences of organised forms of physical activity than their age-related peers (e.g. Ayvazoglu et al., 2015; Harvey et al., 2009; 2014; Johnson & Rosén, 2000; Kim et al., 2011; Stanish et al., 2015; Quesadaa et al., 2018). They tend to drop out of sports early (Johnson & Rosén, 2000) and spend more time doing sedentary activities (Tandon et al., 2019). Lack of physical activity has a major negative impact on both physical and mental health and wellbeing, and therefore the fact that these groups are not engaging is a very important issue to understand and address.

Children's^^[[2]](#footnote-2)^^ engagement in physical activity is significantly influenced by their experiences in various settings, such as physical education (PE), sports, and community based physical activity programmes. Reasons why children diagnosed with ADHD and ASD tend to be less physically active are complex and affected by several different factors, such as safety issues, difficulties with social interaction, being excluded, lack of structure and predictability, lack of enjoyment or self-doubt (Brewster & Coleyshaw, 2011; Coates & Vickerman, 2008; 2010; Shimoni et al., 2010). Previous research by Harvey et al. (2009) showed that boys with ADHD had fewer movement skills than age-related peers, had superficial knowledge about how to preform specific movements, and experienced physical activity in a negative manner. Additionally, a questionnaire by Stanish et al. (2015) indicated that adolescents with ASD enjoyed PE and team sports less and participated in leisure-time sports less frequently compared to their non-diagnosed peers. They also found physical activity hard to learn and did not view organised physical activity as a means of fostering friendships. Furthermore, according to parental reports, safety or unsafety in the surrounding (Hickingbotham, et al., 2021; Blagrave & Colombi-Dougovito, 2019; Gürkan & Kocak, 2023; Siu & Lo, 2020) or lack of adapted suitable physical activity programs (Arnell et al., 2020; Nichols et al., 2019; Papadopoulos et al., 2020) are other factors influencing children’s participation.

Understanding what affects children's physical activity experiences, from micro level (e.g. lack of motor ability) to macro level (e.g. participation opportunities), is crucial for designing health-promoting interventions for children with ADHD and ASD (cf. McLeroy et al., 1988). In the effort to advance knowledge on disabled children's participation in physical activities, scholars have noted a lack of studies on *children’s own experiences* (eg. Fitzgerald et al., 2003; Harvey et al., 2009). However, in the last 10-15 years, more child-centred research has emerged to highlight *children’s perspectives*. Two previous literature reviews on the subject were conducted with children with physical disabilities (Coates & Vickerman, 2008) and students with diverse conditions such as health-related illness, learning disabilities, ASD and sensory disabilities (Haegele & Sutherland, 2015). Both focused on engagement with school-based PE. However, whilst these reviews add important knowledge to the field, they are limited to PE and do not include children with ADHD or have a specific focus on ASD. Moreover, there remains a shortage of synthesized knowledge regarding physical activity experiences of children with neuro developmental disorders. Previous studies demonstrate a significant gap in research, particularly into children’s perspectives. Given the consequences of lack of physical activity for both physical and mental wellbeing, particularly for groups that already face a number of disadvantages and limitations, it is crucial to add a new contribution to existing knowledge. Specifically, there is a need for research involving experiences of children with ADHD and ASD participating in organised physical activity. The purpose of the present study is to carry out a meta-synthesis review of qualitative research to analyse and synthesise empirical research on physical activity experiences among children diagnosed with ADHD and ASD. This will be done by a) creating an overview of previous research; b) synthesizing how previous research describes experiences of organised physical activity among children with ADHD and ASD within the framework of a socio-ecological model; and c) to synthesize positive and negative experiences that are presented in previous research. Before displaying the synthesised result, the theoretical framework and methodological underpinnings will be presented.

## Theoretical and methodological framework

Whilst analysing previous research, it became clear that experiences of organised physical activity among children diagnosed with ADHD or ASD are affected by several factors on diverse levels, from individual factors to societal factors. Inspired by McLeroy et al. (1988)’s framework on diverse factors influencing individual’s health behaviours, I have applied these insights to understand how children's positive and negative experiences in organised physical activity are shaped not solely by their disability, but also by interactions with others and the broader societal context. McLeroy et al. (1988) articulates several levels influencing individual health behaviours within internal and external forces affecting the individual. These can be explained as: *intrapersonal*, *interpersonal*, *institutional*, *community* and *public policy* factors. *Intrapersonal factors* influencing physical activity experiences can include loss of focus, poor movement skills or anxiety, while *interpersonal factors* are related to social relations such as influence by peers or coaches or teachers. *Institutional factors* are tied to the context of specific institutions such as school or a sport club. *Community factors* include the opportunity to join sports programs or adapted sports programs. Finally, *public policy factors* may affect a child’s participation experiences through the application of rules and regulations, for example the integration of children with neuro developmental disorders within mainstream PE (Thoren et al., 2021). Besides McLeroy’s five levels, Sallis et al. (1998) argue for adding *physical/environmental factors* since the physical environment is essential for physical education participation. These maybe equipment or clothes used in the activity, or external factors such as light, temperature and weather. In the analysis, the socioecological model is used, as a synthesising tool, to sort diverse factors influencing children’s experiences.

Further, to enable the child’s perspective to come to the foreground, the paper takes its point of departure in the importance of emphasising children’s perspectives and treating their voices as legitimate study subjects (Fargas-Malet et al., 2010; James, 1998). Therefore, articles focusing on children’s own experiences are at the foreground in this review.

## Material and methods

A qualitative meta-synthesis literature review was conducted (Sandelowski, 2007). This review type is suitable when one wants to retrieve and review results from previous qualitative findings into a synthesised result that generates greater understanding than simply being the sum of each individual research report. Qualitative meta-synthesis includes an interpretative framework and depart from a socio-constructivist paradigm. The reason for synthesizing results from *qualitative studies* is based on the interest in children’s experiences. Qualitative research is a suitable technique to use to explore this.

This meta synthesis offers an integrated description of previous qualitative reports on children with ADHD and ASD and their experiences of participating in organised physical activity. The method includes three steps: 1) a systematic search of qualitative research on the topic; 2) a critical appraisal of the research found according to inclusion criteria; and 3) an interpretative integration of the findings, creating themes and sub-themes (Sandelowski, 2007). These three steps are each described in more detail below.

### Step 1: Systematic search and retrieval of empirical studies

A comprehensive search strategi was used. The literature was searched for in six databases (see Figure 1). These databases were selected to include a mix of sport science, social science and educational research. The search process was conducted in consultation with a librarian who gave feedback on search terms, databases and strategies for searching.

Search words were decided on by using PICo for qualitative studies, including population, phenomenon of interest and context. *Population* was determined as being children and/or adolescents diagnosed with ADHD or ASD. *Interest* was defined as experiences of organised physical activity. Finally, *context* was limited to organised physical activity, i.e. physical activities that are organised and led by a sport coach, leader or PE teacher. This includes, PE, adapted PE, self-contained PE, adapted sports^[[3]](#footnote-3)^, competitive sports and other leader-led physical activities such as community-based physical activity programmes.

The sample was limited to peer reviewed articles, written in English and published between 2010-2021, which was later expanded with literature from 2022-2023^^[[4]](#footnote-4)^^. Starting the search 2010 is motivated by the last 10-15 years of growing body of research that give greater attention to children's voices regarding their experiences in physical activities. The extension of literature was done since these later documents became available after the study had begun.

### Step 2: Critical appraisal of empirical studies

Inclusion criteria were articles on 1) children diagnosed with ADHD or ASD; 2) context of organised physical activity; 3) focus on children’s experiences; 4) empirical studies; 5) qualitative research; and 6) articles written in English. Articles were excluded if they covered adults’ experiences. Further, research on any organised physical activity that made no mention of ADHD, ASD or hidden disability^[[5]](#footnote-5)^ were excluded. Studies on leisure time activities without specifying organised physical activity were also excluded. Articles aiming to evaluate the effect of physical activity as a treatment for ADHD/ASD-symptoms were not included, since the aim of these studies is to explore the effect of the treatment not the experience of the activity.

Articles were first scanned on titles and second on abstracts to determine relevance. A high proportion of the articles were considered non-relevant. This was due to the search process brought up literature on physical activity and children with ADHD or ASD but few of these studies explored *experiences* from the *child’s perspective*. Articles that were deemed relevant were exported to the reference system End Note and checked for duplicates. Articles were then read in full, and any remaining articles not matching the inclusion criteria were excluded. These included articles concerning parent’s perceptions about physical activity participation for children with ADHD and ASD from a parental perspective. A few articles found elsewhere, e.g. references from included articles, were added to the sample. Even though I searched specifically for articles portraying children’s own voices, few articles were found. Two articles were retrospective studies with a focus on childhood experiences, these were included. In total, 18 articles were identified as relevant and were included for a critical appraisal using the the JBI Checklist for Qualitative Research (JBI, 2020) for qualitative research.

Each article was read and appraised by the main author, both individual and collectively. The JBI checklist (JBI, 2020) was used for the individual appraisal. This tool comprises ten questions posed while reading the text, evaluating the congruity between, for example, the methodological perspective and the methods used, as well as the analysis, interpretation, and conclusions drawn. Further aspects, such as researcher influence and ethical considerations, are also taken into account (see further JBI, 2020). After the individual appraisal, one article (Coates & Vickerman, 2010) was excluded as the main findings were based on survey data, and the supplementary focus group interview included only one child with ADHD. It was considered difficult to draw conclusions of relevance to this specific synthesis based on the qualitative parts of this study. The remaining articles were deemed relevant and of satisfactory quality.

To enable a collectively appraisal, each article was read and summarised in a table containing information on author, publication year, title, aim, type of PA, country, sample, method (incl. data generation and analysis), main result, and conclusion. This offered an overview of all articles to be used for further analysis. All included studies had methods, analysis and findings that were relevant for the current literature review. All studies used qualitative methods some was included in a mixed method design (Boucher et al., 2023; Harvey et al 2014 Luymes et al., 2022; Obrusnikova & Cavalier, 2011). In these, the qualitative part was included in the synthesis of results. Several articles used child centered approaches. After the appraisal of each individual study and the collectively appraisal, 17 articles were included in the third step of analyses, i.e. synthesising results. The search process, including the critical appraisal of studies, is visualised in Figure 1, a modified PRISMA flow diagram (Page et al., 2021)

### Step 3: Synthesizing previous research

To answer the first research question, a table was constructed showing characteristics on included studies, such as type of organised physical activity (LTPA or PE), country of research, included research participants and the perspective of the study subjects (retrospective accounts of childhood or contemporary child perspective). See further Table 1, in the result section.

To answer the second and third research questions, the synthesis was conducted through thematic analysis of the data. First, in a deductive process, each included article was read carefully and data extracts from the results sections were sorted by the first author into pre-determined overarching themes based on a socioecological model (McLeroy et al., 1988; Sallis et al., 1998). A data-handling file was used to keep track of the various steps leading to the synthesis of results. Second, in an inductive process, data with similar content were grouped and labelled with key words. Key words representing similar experiences were then developed into sub-themes. For example, the overarching theme “intrapersonal factors influencing experiences”, comprised several sub-themes such as “motor ability and perceived sport specific competence”, “sensory responses” or “emotional responses”, represented by key words and data extracts from each article, including references. For each sub-theme, both positive and challenging experiences were explored. Table 2 illustrates the procedure of thematising the result with some examples.

All articles were not represented in all sub-themes. In table 3 in the result section all themes, sub-themes and references for included articles are presented. Finally, to assess robustness of the synthesized results, each article was scanned once again, checking the content of the article in relation to the sub-themes, making sure that the synthesis was representative for the included articles. Trustworthiness has been achieved by thoroughly working through the data and by checking the credibility of the results against this data.

## Result

### Overview of previous research

The findings of the literature review demonstrate that research within this field has been conducted across various countries, although there is an overweight toward USA (n=6) and Canada (n=6). Most studies were carried out with children diagnosed with ASD (n=12), whereas fewer studies have explored experiences among children diagnosed with ADHD (n=4), or comorbidity of the two (n=1). All five studies in which children with ADHD were included are within the context of LTPA. Further, studies typically involve adolescents, with some also encompassing younger children. Ten studies include both girls and boys; however, it is notable that none of these include many girls. Six studies contain only boys, and one is a case study of one girl and no boys. Interviews are the most used method, and several studies have included prompts such as photos or pictures to improve communication. Table one describes the characteristics of each included study. Organised physical activity has been divided into leisure time physical activity (LTPA), including organised physical activities preformed during spare time and, PE for activities preformed during lessons in school.^[[6]](#footnote-6)^ Articles based on children diagnosed with ADHD have been highlighted in table one so that the reader can see which of the themes/sub-themes adheres to children with ADHD

### Experiences of organised physical activity of children with ADHD and ASD

The result of the synthesis describes physical activity experiences among children diagnosed with ADHD and ASD. The result presents each overarching theme derived from the socio-ecological model, encompassed by various themes focusing on factors influencing experiences and sub-themes that describe children’s experiences within each factor. In each theme, both positive and negative aspects are highlighted.

### Intrapersonal factors influencing experiences

The meta-synthesis demonstrates that ADHD and ASD diagnosed children have mixed experiences – both positive and negative – of organised physical activities, including community-based physical activity programmes, PE, adapted PE, self-contained PE, sports and adapted sports.

#### Motor ability and perceived sport specific competence

The children in Arnell et al. (2018)’s study stated that in order to be able to participate in physical activity they needed a certain minimum level of physical competence. If they perceived that they did not have this, they would probably not participate. Research conducted on children with ADHD/ASD have shown that they display more motor ability difficulties than neurotypical peers (Green, et al., 2009; Harvey et al., 2009, 2014).The sub-theme *lack of motor ability or fitness affecting experiences negatively* includes challenges and bad experiences from participation in organised physical activity (Arkesteyn et al., 2023; Arnell et al., 2018; Harvey et al., 2014; Healy et al., 2013; Obrusnikova & Cavalier, 2011) Such as “Glen” in the study by Harvey et al., (2014, p. 215) saying: ‘Yeah but I don´t like it (racket sports) because I don´t have a lot of precisson, you know’.

The literature review also demonstrates that participants have positive experiences. Children reported that they *experience competence, skill and fitness improvement* when participating in organised physical activity. These positive experiences consist of feelings of competency, shown among ASD-diagnosed children in certain types of physical activities, particularly those to which they were accustomed to and considered to be uncomplicated (Arnell et al., 2018). Further, adolescents with ADHD had specific knowledge of the movements required and perceive that they had the necessary skills for playing sports (Harvey et al., 2012, 2014). This sub-theme also show that some children experience that they are gaining motor ability and fitness through participation in physical activity (Arkesteyn et al., 2023) and that participation in PE/APE developed skills necessary for doing physical activity and exercise (Obrusnikova & Cavalier; Blagrave, 2017).

#### Sensory responses

Sports and physical activity generate various physical stimuli (see further physical factors) that can elicit sensory responses in participants. Research indicates that individuals with neuro developmental disorders, including ADHD and ASD, exhibit heightened sensitivity to sensory stimuli, potentially rendering the environments in which sports and physical activities are conducted particularly challenging. In the reviewed articles, negative responses to stimulus were foremost *auditory and corporal.* Several studies pointed to an overload of sound during physical activity (Arnell, et al., 2018; Haegle & Maher, 2022; Healy et al., 2013; Yessick et al., 2020). Other reactions were corporal, such as disliking the feeling of sweat (Blagrave, 2017; Healy et al., 2013). In a few cases, organised physical activity was described as containing *positive corporal and sensory experiences* such as “feeling heavy” (Blagrave, 2017) or feeling refreshed by taking part in physical activity (Obrusnikova & Cavalier, 2011) as positive feelings. Or as “Andy” explains ‘it ‘feels good’ to have his muscles ‘working hard’ while doing the activity’ (Yessick, et al., 2020, p. 56.). In this sense organised physical activity may contribute to a sense of bodily fulfilment.

#### Emotional responses

Organised physical activity can evoke a wide range of emotions, both positive and negative. A sub-theme presenting positive emotions among children is the *experience of joy and fun* in organised physical activity. Both individual and team activities were found to be enjoyable. Specific individual activities mentioned as positive included gymnastics, trampolining, weightlifting, bowling, bocce ball, and dance (Lamb et al., 2016; Obrusnikova & Cavalier, 2011; Pellerin et al., 2022). Playing games and team sports were also described favourably in some studies, including sports such as baseball and basketball (Boucher, 2023; Lamb et al., 2016; Obrusnikova & Cavalier, 2011; Pellerin et al., 2022). However, children diagnosed with ASD mostly referred to small-sided games, activities with limited and explicit rules, minimal requirements for language interpretation, or restricted aspects of team games –such as shooting a ball at a hoop (Lamb et al., 2016). Furthermore, Arnell et al. (2018) illustrate that play was preferred over more serious team games. Competitive elements were appreciated by some children but experienced as negative by others (Arkensteyn et al., 2023; Arnell et al., 2018). An essential component of enjoyment involved successful engagement in activities (Blagrave, 2017; Pellerin et al., 2022).

Further, positive emotional responses to organised physical activity are captured in the sub-theme *feeling good about oneself* in organised PA (Jachyra et al., 2021), such as participation in LTPA contributing to a ‘good feeling’ (Boucher, 2023) and experiencing a sense of wellbeing (Arnell et al., 2018; Blagrave, 2017). Another sub-theme describes *movement as a calming experience*. Physical activity helped burn excessive energy (Lee et al., 2014), was described as an emotional venting or outlet (Jachyra, et al., 2021; Lee et al., 2014), or a respite from stress (Arnell et al., 2018). These examples illustrate the significant role that physical activity can play in the positive emotional experiences among children and adolescents with ADHD and ASD.

Unfortunately, children also associated several negative feelings with organised physical activities, explained in the sub-theme *feeling bored and disengaged.* Harvey et al., (2012; 2014) found that some children diagnosed with ADHD showed a dislike of leisure time physical activities, including experiences of finding physical activity too demanding. Similar results were found among children diagnosed with ASD (Healy et al., 2013; Arnell, et al., 2017). Further, activities were perceived as uninteresting (Jachyra et al., 2021; Obrusnikova & Cavalier, 2011). Some specific elements were also found to be boring, such as dance (Arnell et al., 2018; Luymes et al., 2022) and, for some, competitive activities (Arnell et al., 2018).

Physical activity also generated *anxiety and worrying about participation* (Arkesteyn et al., 2023; Arnell et al., 2018; Jachyra et al., 2021), and in addition, more specific types of anxiety were found, such as performance anxiety (Harvey et al., 2014), body appearance-anxiety (Arnell et al., 2018), or fear of getting hurt (Boucher, 2023: Healy et al., 2013). Participation in organised physical activities sometimes led to *anger and frustration*, directed both at others and at the children themselves (Ing & Mills, 2017).

#### Motivation and self-esteem

Motivation is a crucial facilitator for engaging in sports (Obrusnikova & Cavalier, 2011). Physical activity became motivating for the participating children when they perceived a *sense of meaning* by participating in activities. This was experienced when activity was perceived as important – and when they were associated with a goal or health benefits, such as gaining health (Harvey et al., 2014) or mental health (Blagrave, 2017) or doing physical activity with an intention to to stay fit (Arkesteyn et al., 2023). Also, fear of ill-health or obesity served as a motivator for engaging in health-enhancing activities (Arnell et al., 2018). Other aspects that brought meaning to the activity was to achieve results in sports (Arkesteyn et al., 2023). Further, autonomy and freedom of choice enhanced motivation to participate in activities (Arkesteyn et al., 2023; Arnell, et al., 2018). Additionally, participation in organised physical activity were experienced as helping to *grow self-esteem* (Arnell et al., 2018; Blagrave, 2017).

Further, the synthesis show that *low motivation makes participation challenging* (Arkesteyn et al. (2023; Arnell et al., 2018). Both studies identified a lack of motivation to engage in physical activity among children with ASD. Even when activities were deemed enjoyable, some children in the study by Arnell et al. (2018) found it difficult to initiate physical activity. Moreover, experiences of *self-doubt when participating* in organised PA, was found among children with ADHD (Ing & Mills, 2017) and a lack of confidence, feelings of insecurity, and low self-esteem was found among children with ASD (Arnell et al., 2018).

#### Challenges in executive functions

ADHD and ASD are associated with disability-specific constraints, some of these are challenges in executive functions. In the reviewed research articles, some of these constraints are described in relation to their contributions to negative experiences of organised physical activity. First, *challenges caused by inattentiveness* in organised physical activity, were described as ‘drifting off’ and caused difficulties in following instructions (Haegele & Maher, 2023; Lee et al., 2014).

*Lack of control* can caused difficulties especially among children with ADHD, which is described in studies by Ing & Mills (2017) and Lee et al. (2014). Impulsive behaviour, such as blurting out comments (Lee et al., 2014) to other children or deliberately tackling someone in a ball game (Ing & Mills, 2017), caused difficulties for some children. Lee et al. (2014) gives an example of this with a statement from “Michael”: ‘*I blurted out a comment. I said something about him like just getting stupider and stupider. And I stopped and I was like ‘Why would I say that’?’* (p. 350). Children diagnosed with ASD also portrayed how lack of control, such as biting or hitting other participants prevented them from playing sports (Boucher, 2023).

### Interpersonal factors influencing experiences

Friends and family, as well as teammates and coaches are important in shaping both negative and positive experiences of organised physical activity.

*Significant others*

Significant other’s such as family and friends, play a crucial role in influencing participation in, and experiences of, physical activity. The sub-theme *family and friends as important LTPA partners and supporters* indicates the necessity of having significant others facilitate engagement in PA during leisure time (Arnell et al., 2018; Blagrave, 2017; Boucher, et al., 2023; Harvey et al., 2014; Jachyra et al., 2021; Obrusnikova & Cavalier, 2011). Conversely, lacking companions to participate with or lack of parental support can hinder participation in LTPA (Arkesteyn et al., 2023; Obrusnikova & Cavalier, 2011).

*Peer relations*

According to Healy et al. (2013, p. 41), PE ‘can socially benefit children with ASD’. Positive experiences seem to occur when the child feels accepted (Ing & Mills, 2017) and experiences friendship in the team or group (Arkesteyn et al., 2023; Healy et al., 2013; Luymes, et al., 2022; Yessick et al., 2020). This is captured in the sub-theme *sense of belonging* in organised PA and is exemplified by Haegele & Maher (2022)’s study in which one student (“Chris”) noted developing strong relationships with his classmates in PE, which he attributed to his ability as an athlete and a shared sense of group athleticism. Further, sharing a common goal (Lee, 2014) and working as a team (Harvey et al., 2014), are shown to be important to develop and keep friendship going. In a study of self-contained PE by Pellerin et al. (2022), a sense of belonging was identified as the importance of gaining friendships with other children with disabilities.

In contrast, the sub-theme *not feeling at ease in the group* captures challenges in peer relations, such as the feeling of being in an unpleasant group (Arkesteyn et al., 2023) or experiencing difficulties communicating to, or adjusting to other children (Arkesteyn et al., 2023; Arnell, et al., 2018). This may lead children to reduced involvement in team sport or group activities (Arnell, et al., 2018). Haegele & Maher (2022) identify that a barrier to forming friendships in PE is the lack of shared interests, i.e. where autistic students found themselves having different interests compared to their peers. In the most severe cases of negative peer relations, children were *subjected to bullying* from other children, primarily through verbal means, but in some cases, physically (Haegele & Maher, 2022; Healy et al., 2013; Jachyra et al., 2021). Another form of bullying is exclusion by other children, such as singling out the child with a disability (eg. being selected last for a group or not being included in play or games). This also involved situations where the children were perceived as lacking ability, and other children verbally accused them for not being able to play the game correctly, — for example, as “Bill” expressed: ‘They keep saying that ‘Bill can’t catch a ball.’’ (Healey et al., 2013, p. 225).

Another issue concerns the impact of negative social comparisons with peers, described in PE. Such comparisons may relate to performance, or to perceiving other children’s competitiveness as distressing. Some children with ASD described feeling less energetic than their peers and reported feeling bad when they came last in competitions (Healy et al., 2013). Other social comparisons were related to physical appearance, such as low perceived body satisfaction, particularly during situations like changing clothes or showering in PE (Arnell et al., 2018). Furthermore, children in Arnell et al’s study described feeling uneasy when under scrutiny or evaluation by others, even when the assessment was positive.

*Coach/teacher relations*

Results of the literature review shows that *appreciation of a caring and responsive coach/teacher* was important to experience organised physical activity in a positive manner (Arkesteyn et al., 2023; Arnell et al., 2017; Blagrave, 2017; Boucher, 2023; Ing & Mills, 2017; Lee, et al., 2014; Yessick, et al., 2020). Among children included in the studies, several had positive experiences of their PE/adapted- och self-contained PE teacher (Arkesteyn et al., 2023; Arnell et al., 2017; Blagrave, 2017; Boucher, 2023; Yessick, et al., 2020). As an example, Blagrave (2017) cites children feeling that the PE teacher was helpful and friendly. An important aspect highlighted by the children was to have a teacher that was ‘keen, responsive and clear, besides being understanding of their abilities and needs’ (Arnell et al., 2018, p. 1798). Similar results are found in after-school activities such as sports. Research by Lee, et al. (2014) indicate that children with ADHD who felt that coaches were supportive and worked patiently with them made them feel positive about taking part in sport. The importance of a caring and understanding coach is also evident in the retrospective story by Ing & Mills (2017). The authors stress the need of coaches to ‘remain patient and develop the trust and respect required to make athletes feel competent and supported’ (p. 7).

Unfortunately, not all relationships were experienced as positive. Challenges in *interactions between coaches and athletes, or teachers and students*, were primarily addressed in LTPA (Arkesteyn et al., 2023; Jachyra et al., 2021); Lee, et al., 2014). A study by Lee et al. (2014) found that children with ADHD perceived emotional reactions from coaches, such as frustration or anger, negatively. For instance, athletes reported adverse experiences when coaches responded negatively to their mistakes. Another challenge relates to children’s distractibility (cf. *executive functions*), and the redirection they receive from teachers or coaches, which has been reported to result in negative experiences (Lee et al., 2014; cf. Blagrave, 2017, for APE). Children also encountered difficulties when coaches failed to adequately consider their needs. For instance, Jachyra et al. (2021) describe how excessive verbal instructions from coaches can make it challenging for children who have greater difficulties with many instructions. There are also cases of exclusion from teachers or leaders in sports. In PE, examples are show to how the PE teacher directs the child to do something else while for example playing a ball game. In sports, a child may be sent away from the playing field due to incidents occurring such as tackling another player unfairly – this is experienced as exclusion from the child’s point of view (Ing & Mills, 2017).

## Institutional factors influencing experiences

Physical activities are organised in different arenas such as schools, sports clubs, and community based physical activity centres, each which may offer unique experiences. Few articles described experiences of institutional factors, those that did are captured in the theme *Levels of structure, predictability and adaption offered by the institution*. The positive aspects of this theme are exemplified in Arkesteyn et al. (2023), where children express that participating in LTPA held at the same location and at the same time was beneficial for their engagement. Similarly, performing familiar activities in a familiar setting was described as a positive experience according to Arnell et al. (2018) and having consistent routines and visual targets and trackers was experienced as positive in PE (Yessick et al, 2020). In essence, physical activity preformed in an institution providing safety through routines and familiar settings are experienced positive. In contrast, a lack of predictability was regarded as negative (Arnell et al., 2018).

In two articles, PE was specifically considered to be an institution with *lack of adaptability*, by being perceived as both unpredictable and inflexible. Some children found lack of adaptions to their needs in PE, which in turn limited their participation (Arnell et al., 2018). Further, according to children diagnosed with ASD, PE was experienced as negative due to rigidity, restricting freedom of choice in activities and, lack of influence over who to do an activity with (Arnell, et al., 2018). Lack of adaptability in PE, may lead to self-exclusion by the child. In some cases, children choose to self-exclude by not wanting to participate in some activities in PE (Healy 2013) or to request removal of PE classes from their individual education plan (Arnell, et al., 2018).

Community and Public-policy factors influencing experiences

Few of the included studies provide information on how children perceive community and public-policy factors as influencing their experiences of organised physical activity. When such factors are mentioned by children, they tend to relate to experiences of *opportunity and availability* to participate in organised activities. Access to appropriate leisure-time LTPA was described as positive and seen as a facilitator of participation (Arkesteyn et al., 2023; Obrusnikova & Cavalier, 2011). However, several children reported a lack of opportunity and availability, including barriers such as a shortage of adapted sport activities (Boucher, 2023), activities being too time-consuming or costly (Harvey et al., 2014), and a lack of transportation to and from suitable activities (Arnell et al., 2018; Obrusnikova & Cavalier, 2011).

*Hindering policies or regulations* may act as a barrier to participating in organised physical activity or negatively influence the experience. Jachyra, et al. (2021) demonstrate exclusion from activities of interest due to service providers requiring a personal support worker for participation. In cases where this support was unavailable, children were unable to join these activities. In a study by Arkesteyn et al. (2023) some children considered the mandatory aspect of PE as negative. Furthermore, dressing rules and rules concerning transportation to and back from the PE gym were experienced as negative.

Physical factors influencing experiences

*The outdoor environment*

*Weather and temperature* had an *impact on participation and shaped negative experience* of physical activity in the outdoor environment. Temperature (e.g. too hot or too cold) as well as weather (e.g. wind, rain or snow) had a negative and limiting effect, or excluded participation altogether (Arkesteyn et al., 2023; Arnell, et al., 2018; Obrusnikova & Cavalier, 2011). Similar results were found in a questionnaire by Stanish et al. (2015) showing that 81% of children diagnosed with ASD reported that, in their opinion, it was sometimes too hot or cold to do physical activity and most of these reported that this hindered them from partaking. Likewise, *bugs* in the outdoor surroundings may hinder children's participation or negatively affect their experience (Obrusnikova & Cavalier, 2011, Arnell, et al., 2018). Only one study acknowledges positive experiences of the outdoor context, in which being outside and expiring nature was highlighted as positive (Arkensteyn, 2023).

*The indoor environment*

*Adequate space and a pleasant gym environment* to do physical activity in, is highlighted in some of the studies. On the positive side, some children in a study by Arkesteyn et al. (2023) talked about experiencing a spacious and quiet gym and that this facilitated their participation in PE. The PE gym was also described as a relief from other negative sensory stimulus such as bright light in the classroom in a study by Blagrave (2017). Children in Lamb et al’s (2016) study made positive associations with the PE teacher's office when talking about spaces for PE. Apart from these studies, most studies acknowledge the indoor environment as less pleasant.

Physical aspects like *too much noise make experience unpleasant*. The physical activity context is generally experienced as loud or noisy (Arkesteyn, 2023; Arnell et al., 2018; Harvey et al., 2014). For instance, a boy in Arnell et al. (2018)’s study describes dancing as a loud activity that made him tired and unable to focus. Or as “Greg” a child with ADHD perceived sports participation: ‘Sometimes there are parents that are screaming and it´s hurting my ears’ (Harvey et al., 2014, p. 215).

In Arnell, et al. (2018) and Lamb et al. (2016) articles, *changing rooms* were specifically described as a *chaotic and unpleasant area*, evoking negative feelings associated with participating in PE. The corridor leading up to the PE gym was also experienced as crowded and noisy (Lamb et al., 2016). Further, Haegele & Maher (2022) emphasise the changing room as an especially problematic physical space due to occurrence of bullying.

Lastly, as children with ADHD and ASD appear to have an increased fear of getting hurt, the feeling of unsafety in the environment was sometime driven by physical factors such as equipment perceived as dangerous (Arkesteyn et al., 2023; Harvey et al., 2014; Healy et al., 2013; Obrusnikova & Cavalier, 2011).

## Discussion of synthesised research

The research overview presented in Table 1, demonstrates that there is an overall lack of research into experiences of organised physical activity among the studied population, from children’s own perspective and, few of the studies included children diagnosed with ADHD. Further, girls are frequently underrepresented both in studies on ADHD and ASD. It can thus be stated that we specifically need more knowledge about experiences among ADHD diagnosed children and further, advance knowledge on ADHD/ASD diagnosed girls’ experiences of organised physical activity.

Research is produced in several countries, with a greater proportion of the research is in USA and Canada whereas there is a lack of studies in South America, Asia and Africa. Further, studies in the Nordic countries are scarce, with only one Swedish study included.

The synthesis demonstrates that children have both positive and negative experience of organised physical activity. These experiences are shaped by intrapersonal factors, such as their individual needs and abilities, interpersonal factors, including their relationships with others, and various external factors at the institutional, community or policy level. The latter often pertains to the availability and adaptation of activities, with institutional factors specifically relating to the extent of accommodations made for individual needs. Interestingly, PE was portrayed in some studies as an institution lacking adaptability, while in others it was depicted as offering structure and predictability. This suggests the need for further studies on how institutionalised aspects of PE affect experiences. Given that intrapersonal and interpersonal factors are more immediate to the child and frequently cited in descriptions of their experiences, the discussion will primarily focus on these factors. The results will further be compared with other relevant studies, and implications from the literature review will be discussed.

In understanding the research, it is important to consider how experiences of physical activity may be impacted by the nature of ADHD and ASD, and how these shape the lives and reactions of those with those conditions. For instance, certain intrapersonal factors that negatively influence experiences of organised physical activity may be considered disability-specific, such as challenges related to executive functioning. Inattention was challenging, especially in relation to following instructions. Impulsivity caused difficulties for some when interacting with others and may in turn cause challenges in peer relations due to the child not acting as expected by others (eg. blurting out comments). These results are consistent with parental reports suggesting that disability-specific constraints may pose additional challenges for children with ADHD or ASD (Ayvazoglu et al., 2015; Blagrave & Colombo-Dougovito, 2019; Gürkan & Kocak, 2020; McMahon et al., 2020). Further, as explained at the beginning of the article, research show that motor difficulties are more common among children with neuro developmental disorders (Green, et al., 2009; Harvey et al., 2014). In the analysed articles, lack of motor ability or fitness caused difficulties in participating in activities and, for some, generated feelings of inadequacy. Another negative experience was related to sensory responses, with difficulties in tolerating sweat, noise or bright light. High sensitivity to stimuli is common in children with ASD (Yessick et al., 2022). The synthesis aligns with other research suggesting that the disability itself may pose additional challenges in participation in activities (compare Arnell, et al., 2020; Nichols et al., 2019). Based on these results, it is important that coaches/teachers are aware of the challenges that may be specific to children with ADHD and ASD and potential strategies and solutions, to enable them to adjust their approach to reflect children’s needs.

Furter, participation in physical activity provoked negative feelings of boredom, stress and anxiety among some of the children, as well as low self-esteem and lack of motivation (cf. Hickingbotham, et al., 2021). This implies that coaches/teachers need to ensure physical and mental support for children. Drawing on research by Jones &Thomas (2015), and the term “pedagogical scaffolding”, I suggest that scaffolding could help children with ADHD/ASD to feel safe, motivated and develop self-confidence while learning through sports with the support of the coach. I base this suggestion on the view of scaffolding as a mediator in the learning process taking into consideration both the context in which learning is takin place (macro and meso level) and the actual interaction within this context (micro level).

On the positive side, several children enjoyed physical activity and experienced joy as a result of their participation. Although many children perceive motor difficulties as a hindrance, the literature review indicates that organised physical activity can enhance motor skills and boost confidence, enabling participation in other activities. Furthermore, some studies suggest that such activities can improve self-esteem and highlight the importance of motivation for children's participation. These findings align with parental reports suggesting that sport activities contribute to development in various life aspects, both within and beyond the realm of sports (Gürkan & Kocak, 2020; Kristén, et al., 2023; May et al., 2018; Rodriquez et al., 2022). Based on the review, it is importance to include and to keep children with ADHD and ASD in organised activities so that it can help them improve their abilities and confidence well enough to promote participation in sports and PE.

The synthesis further highlights how interpersonal relations may cause difficulties for ADHD and ASD diagnosed children. Results demonstrate negative experiences due to lack of adaption among coaches and teachers. Also, when coaches or teachers reacted emotionally or in frustration at children’s behaviour, children found it difficult to bear. This result is in line with research on parent’s perceptions about coaches, which highlight the challenges of lack of adaption and greater use of intimidating coaching among coaches working with hyper-active children (Vargas, et al., 2019). Further, other research demonstrate that coaches lack experience, knowledge and practice on training children with neuro developmental disabilities (Ayvazoglu et al., 2015; Blagrave & Colombo-Dougovito, 2019; Gregor et al., 2018; Gürkan & Kocak, 2020; Vargas et al., 2015). On the positive side, some of the children in the research reports talked positively about their coaches and teachers and found them kind and caring. A supportive coach-athlete/teacher-student relation was indicated as important for children with ADHD and ASD. Similar results are shown in a study of children with physical disability in the school subject PE. The importance of a strong relationship between PE teacher and child were demonstrated as a positive factor to participate (Fitzgerald, 2005).

Additionally, some research indicates that children experienced exclusion and even bullying from their peers. This aligns with findings on leisure activities (Brewster & Coleyshaw, 2011) and PE experiences among children with other disabilities or special educational needs (Coates & Vickerman, 2008; Haegele & Sutherland, 2015; Fitzgerald, 2005; Fitzgerald & Stride, 2012). Further, social comparison was perceived negatively by the participating children, similar to findings in a study on children with disabilities in PE (Fitzgerald, 2005). However, not all interpersonal experiences are negative. Although previous research has shown that children with ASD are less inclined than typically developed children to view organised physical activity as a way to make friends (Stanish et al., 2015), this literature review indicates that sports activities or (adapted) physical education in school can facilitate friendships for individuals with ADHD and ASD (Healey et al., 2013; Harvey et al., 2014; Lee, et al., 2014). Similar results were found in an earlier study by Harvey et al. (2009) and in parental reports (Gürkan & Kocak, 2020). Based on these findings, it is important that activities encourage cooperation and inclusiveness to help children form friendships through organised physical activity.

A limitation of this study is the difficulty in identifying qualitative research that provides a firsthand perspective from children diagnosed with ADHD and ASD. Several records did not meet the inclusion criteria, which raises the possibility that relevant studies may have been overlooked due to the search terms used. It is conceivable that alternative search terms could have resulted in the inclusion of additional articles. However, it is important to note that multiple search terms were tested prior to finalising the selected terms, and these did not yield any records beyond those identified through the chosen terms. Furthermore, a librarian was consulted to ensure the most effective search strategy was employed.

Furthermore, the two overarching themes most frequently addressed in the included research articles are intra- and interpersonal factors influencing experiences of organised physical activity. Within these themes, all sub-themes (though not all individual codes) included research involving both study populations. However, it should be noted that research focusing on the experiences of children with ADHD remains limited, and firm conclusions cannot be drawn. Moreover, none of the overarching themes related to institutional factors or community and policy factors influencing experiences were represented in studies involving children diagnosed with ADHD. This may reflect a lack of research attention to these areas. Furthermore, although ADHD and ASD are often comorbid, they are heterogeneous conditions. Even within each diagnosis, children may have diverse perceptions of their condition and how it affects them (Ringer, 2020). Efforts were made to present the results in a way that clearly indicates whether findings apply to one or both diagnoses. This distinction is also reflected in Table 3, where references from articles focusing on ADHD are highlighted. Further, it should be acknowledged that some studies have limited inclusion to children with an IQ over 70 and/or sufficient verbal abilities to engage in interviews. While this enhances the credibility of the findings, as participants are better able to articulate their experiences, it also narrows the total scope of the findings. The exclusion of children with more significant cognitive or communicative challenges means that important perspectives are missing, which limits the transferability and richness of the insights. This highlights the need for further research that includes children with a wider range of communicative and cognitive profiles, to develop a more inclusive understanding of their experiences.

The term “organised physical activity”, further encompasses various forms, including PE in schools (both adapted, self-contained and integrated), community based physical activity centres, and sports. Despite this diversity, research indicates that many experiences are similar across these different activities. When I have found it important, I have specified the context from which each result originates to ensure clarity. The context of PE and LTPA is also indicated in Table 3 to ensure that the reader can identify which type of activity each theme or sub-theme relates to. Notably, none of the studies focusing on children diagnosed with ADHD included experiences of the school subject PE.

A further limitation is the high representation of reports from USA and Canada which may affect the generalisability of the overall result. Readers should keep in mind that physical activity contexts may be different in other countries. One last limitation is that a qualitative meta-synthesis although helping to get a fuller picture of a phenomenon also includes a reinterpretation of research. As such, the result presented are ‘two stages away from the individual’s own words’ (Ringer, 2020, p.), meaning that there is a risk of losing part of the experiences expressed by the participating children. To limit this risk, I have given several examples from the original articles to strengthen the interpretations made, while at the same time, keeping close to the research that the synthesis builds upon. I have also, when appropriate, included some original quotes to provide examples of and emphasise the children’s own voices.

Conclusion and further implementations

To conclude, the synthesis of qualitative studies highlights the diversity of experiences among individual children with ADHD and ASD in relation to organised physical activity. The results highlight specific aspects that can inform further research and efforts to include children with ADHD and ASD in various forms of organised physical activities.

Although several studies were found in total on physical activity and children diagnosed with ADHD or ASD, only a few explored children’s own *experiences*. There is a particular lack of studies including children with ADHD and girls with both ADHD and ASD. As a researcher at a Swedish university, I recognise the need for more research within a Swedish/Nordic context. Overall, there is a need for more research that incorporates first-hand perspectives from the children themselves.

Since ADHD and ASD are common disabilities among children (with ADHD affecting 5-8% and ASD 1%), it is crucial for their physical and mental health and well-being that they do not miss out on the benefits of organised physical activity. This highlights the need for teachers, leaders, and coaches to acquire more knowledge on how to support these children. Understanding what matters to them can help inform institutions such as schools and sports organizations on how to empower children in and through organised physical activities. The findings clearly show that children have valuable insights that stakeholders in organised physical activities must consider. It is essential that this information is heard and acted upon to create inclusive and supportive environments for all children.

**Tables and figures** (submitted separately):

Figure 1: Modified PRISMA flow diagram

Table 1. Information on included studies

Table 2. Different aspects in children’s experiences of PA

Table 3: Thematization of factors influencing experiences of organised physical activity among children with ADHD and ASD and, experiences within these factors.

**Contributions statement**:

Conceptualization, data curation, formal analysis, funding acquisition, investigation, methodology, project administration, resources, validation and writing the original draft was done by Karin Grahn.

**Disclosure statement:** No conflict of interest.

**Funding details:** This work was supported by the Swedish Research Council for Sport Science

[number: P2022-0042].

**Data availability statement:** all included articles are published online.

**Acknowledgements**: Since English is not my first language I have used ChatGPT-4the in the writing process to improve language. Any suggestion for language editions have been checket thoroughly to make sure that the content have not ben altered.

**Biographical note:** *Karin Grahn* is a senior lecturer at the Department of Food and Nutrition, and Sport Science, University of Gothenburg. She is teaching in the Sports Coaching programme and has research interests in the fields of youth sports, neurodevelopmental disabilities, gender, and sports coaching. Karin has specialised in using qualitative methods and analysis and have conducted several empirical studies using interviews, focus groups, participatory observations, text and disscourse analysis.

## References

Arkesteyn, A., Cornelissen, V., Steyaert, J., Vancampfort, D., & Van Damme, T. (2023). Barriers and facilitators of physical activity participation in adolescents with autism. *Children’s Health Care*, *ahead-of-print*(ahead-of-print), 1–44. <https://doi.org/10.1080/02739615.2023.2228693>

American Psychiatric Association (2013). *Diagnostic and statistical manual of mental disorders DSM-5* (5th ed.). Arlington, VA : American Psychiatric Association.

Arnell, S., Jerlinder, K., & Lundqvist, L.-O. (2018). Perceptions of Physical Activity Participation Among Adolescents with Autism Spectrum Disorders: A Conceptual Model of Conditional Participation. *Journal of Autism and Developmental Disorders*, *48*(5), 1792-1802. <https://doi.org/10.1007/s10803-017-3436-2>

Arnell, S., Jerlinder, K., & Lundqvist, L.-O. (2020). Parents’ perceptions and concerns about physical activity participation among adolescents with autism spectrum disorder. *Autism : the international journal of research and practice*, *24*(8), 2243-2255. <https://doi.org/10.1177/1362361320942092>

Ayvazoglu, N. R., Kozub, F. M., Butera, G., & Murray, M. J. (2015). Determinants and challenges in physical activity participation in families with children with high functioning autism spectrum disorders from a family systems perspective. *RESEARCH IN DEVELOPMENTAL DISABILITIES*, *47*, 93-105. <https://doi.org/10.1016/j.ridd.2015.08.015>

Blagrave, A. J., & Colombo-Dougovito, A. M. (2019). Experiences Participating in Community Physical Activity by Families with a Child on the Autism Spectrum: a Phenomenological Inquiry [Article]. *Advances in Neurodevelopmental Disorders*, *3*(1), 72-84. <https://doi.org/10.1007/s41252-018-0094-0>

Blagrave, J. (2017). Experiences of children with autism spectrum disorders in adapted physical education. *European Journal of Adapted Physical Activity*, *10*(1), 17-27. <https://doi.org/10.5507/euj.2017.003>

Boucher, T. Q., McIntyre, C. L., & Iarocci, G. (2023). Facilitators and barriers to physical activity involvement as described by autistic youth with mild intellectual disability. *Advances in Neurodevelopmental Disorders, 7*(4), 512–524. <https://doi.org/10.1007/s41252-022-00310-5>

Brewster, S., & Coleyshaw, L. (2011). Participation or exclusion? Perspectives of pupils with autistic spectrum disorders on their participation in leisure activities. *British Journal of Learning Disabilities*, *39*(4), 284-291. <https://doi.org/10.1111/j.1468-3156.2010.00665.x>

Coates, J., & Vickerman, P. (2008). Let the children have their say: children with special educational needs and their experiences of Physical Education - a review. *Support for learning*, *23*(4), 168-175. <https://doi.org/10.1111/j.1467-9604.2008.00390.x>

Coates, J., & Vickerman, P. (2010). Empowering children with special educational needs to speak up: experiences of inclusive physical education. *Disability and rehabilitation*, <https://doi.org/10.3109/09638288.2010.497037>

Fargas-Malet, M., McSherry, D., Larkin, E., & Robinson, C. (2010). research with children: methodological issues and innovative techniques. *Journal of early childhood research : ECR*, *8*(2), 175-192. <https://doi.org/10.1177/1476718X09345412>

Fitzgerald, H. (2005). Still feeling like a spare piece of luggage? Embodied experiences of (dis)ability in physical education and school sport. *Physical Education and Sport Pedagogy*, *10*(1), 41–59. <https://doi.org/10.1080/1740898042000334908>

Fitzgerald, H., Jobling, A., & Kirk, D. (2003). Valuing the Voices of Young Disabled People: Exploring Experience of Physical Education and Sport. *European journal of physical education*, *8*(2), 175-200. <https://doi.org/10.1080/1740898030080206>

Fitzgerald, H., & Stride, A. (2012). Stories about Physical Education from Young People with Disabilities. *International Journal of Disability, Development, and Education*, *59*(3), 283–293. <https://doi.org/10.1080/1034912X.2012.697743>

Green, D., Charman, T., Pickles, A., Chandler, S., Loucas, T. O. M., Simonoff, E., & Baird, G. (2009). Impairment in movement skills of children with autistic spectrum disorders. *Developmental medicine and child neurology*, *51*(4), 311-316. <https://doi.org/10.1111/j.1469-8749.2008.03242.x>

Gregor, S., Bruni, N., Grkinic, P., Schwartz, L., McDonald, A., Thille, P., Gabison, S., Gibson, B. E., & Jachyra, P. (2018). Parents' perspectives of physical activity participation among Canadian adolescents with Autism Spectrum Disorder. *RESEARCH IN AUTISM SPECTRUM DISORDERS*, *48*, 53-62. <https://doi.org/10.1016/j.rasd.2018.01.007>

Gürkan, R. K., & Kocak, F. (2020). Perceived constraints and facilitators of participation in physical activity by individuals with autism spectrum disorders [Article]. *Physical Activity Review*, *8*(1), 51-63. <https://doi.org/10.16926/par.2020.08.07>

Gürkan, R. K., & Kocak, F. (2023). Double punch to the better than nothing: physical activity participation of adolescents with autism spectrum disorder. *INTERNATIONAL JOURNAL OF DEVELOPMENTAL DISABILITIES*, *ahead-of-print*(ahead-of-print), 1-13. <https://doi.org/10.1080/20473869.2021.2009636>

Haegele, J. A., & Maher, A. J. (2023). A creative non-fiction account of autistic youth integrated physical education experiences. *Disability & Society*, *38*(9), 1647–1666. <https://doi.org/10.1080/09687599.2021.2007361>

Haegele, J. A., & Maher, A. J. (2022). Male autistic youth experiences of belonging in integrated physical education. *Autism, 26*(1), 225–234. <https://doi.org/10.1177/13623613211018637>​

Haegele, J. A., & Sutherland, S. (2015). Perspectives of Students with Disabilities Toward Physical Education: A Qualitative Inquiry Review. *QUEST*, *67*(3), 255-273. <https://doi.org/10.1080/00336297.2015.1050118>

Harvey, W. J., Wilkinson, S., Pressé, C., Joober, R., & Grizenko, N. (2012). Scrapbook interviewing and children with attention-deficit hyperactivity disorder. *Qualitative Research in Sport, Exercise and Health*, *4*(1), 62–79. <https://doi.org/10.1080/2159676X.2011.653497>

Harvey, W. J., Reid, G., Bloom, G. A., Staples, K., Grizenko, N., Mbekou, V., Ter-Stepanian, M., & Joober, R. (2009). Physical activity experiences of boys with and without ADHD. *Adapted Physical Activity Quarterly*, *26*(2), 131-150. <https://doi.org/10.1123/apaq.26.2.131>

Harvey, W. J., Wilkinson, S., Pressé, C., Joober, R., & Grizenko, N. (2014). Children Say the Darndest Things: Physical Activity and Children with Attention-Deficit Hyperactivity Disorder. *Physical Education and Sport Pedagogy*, *19*(2), 205-220. <http://dx.doi.org/10.1080/17408989.2012.754000>

Healy, S., Msetfi, R., & Gallagher, S. (2013). 'Happy and a bit Nervous': the experiences of children with autism in physical education. *British Journal of Learning Disabilities*, *41*(3), 222-228. <https://doi.org/http://dx.doi.org/10.1111/bld.12053>

Hickingbotham, M. R., Wong, C., & Bowling, A. (2021). Barriers and facilitators to physical education, sport, and physical activity program participation among children and adolescents with psychiatric disorders: A systematic review. *Translational Behavioral Medicine, 11*(5), 1048–1057. <https://doi.org/10.1093/tbm/ibab050>

Ing, C., & Mills, J. P. (2019). “Hey, look at me”: An {auto}ethnographic account of experiencing ADHD symptoms within sport. *Qualitative Research in Sport, Exercise and Health*, *11*(2), 274–283. <https://doi.org/10.1080/2159676X.2017.1405362>

Jachyra, P., Renwick, R., Gladstone, B., Anagnostou, E., & Gibson, B. E. (2021). Physical Activity Participation among Adolescents with Autism Spectrum Disorder. *Autism: The International Journal of Research and Practice*, *25*(3), 613-626. <https://doi.org/10.1007/s10803-017-3436-2>

James, A. (1998). *Theorizing childhood*. Cambridge New York : Polity Press : Teachers College Press.

JBI. (2020). *Checklist for Qualitative Research*. Joanna Briggs Institute. Retrieved from: https://jbi.global/critical-appraisal-tools

Jones, R. L., & Thomas, G. L. (2015). Coaching as “scaffolded” practice: further insights into sport pedagogy. *Sports Coaching Review*, *4*(2), 65–79. <https://doi.org/10.1080/21640629.2016.1157321>

Johnson, R. C., & Rosen, L. A. (2000). Sports behavior of ADHD children. *Journal of attention disorders*, *4*(3), 150-160. <https://doi.org/10.1177/108705470000400302>

Kristén, L., Nyholm, M., & Lydell, M. (2023). Parents’ experiences of their child with neurodevelopmental disorders participating in a physical activity on prescription (PAP) intervention. *European Journal of Adapted Physical Activity*, *16*. <https://doi.org/10.5507/euj.2022.009>

Kim, J. B., Mutyala, S., Agiovlasitis & Fernhall, B. (2011). Health behaviors and obesity among US children with attention deficit hyperactivity disorder by gender and medication use. Preventive medicine, *52*(3), 218-222. <https://doi.org/10.1016/j.ypmed.2011.01.003>

Lamb, P., Firbank, D., & Aldous, D. (2016). Capturing the world of physical education through the eyes of children with autism spectrum disorders. *Sport, Education & Society*, *21*(5), 698-722. https://doi.org/10.1080/13573322.2014.941794

Lee, H., Dunn, J. C., & Holt, N. L. (2014). Youth Sport Experiences of Individuals With Attention Deficit/Hyperactivity Disorder. *Adapted Physical Activity Quarterly*, *31*(4), 343-361. <https://doi.org/10.1123/apaq.2014-0142>

Luymes, N. J., Bryden, P. J., & Fletcher, P. C. (2022). "I Think She's Found Her Groove": Evaluating A Young Girl's Involvement in an Inclusive Physical Activity Program. *Palaestra (Macomb, Ill.)*, *36*(2), 5-13.

May, T., Rinehart, N., Barnett, L., Hinkley, T., McGillivray, J., Skouteris, H., Stephens, D., & Goldfinch, D. (2018). “We’re doing AFL auskick as well”: Experiences of an adapted football program for children with autism. *Journal of Motor Learning and Development*, *6*(1), 130–146. <https://doi.org/10.1123/jmld.2016-0055>

McLeroy, K. R., Bibeau, D., Steckler, A., & Glanz, K. (1988). An Ecological Perspective on Health Promotion Programs. *Health education quarterly*, *15*(4), 351-377. <https://doi.org/10.1177/109019818801500401>

McMahon, J., Wiltshire, G.-E., McGannon, K. R., & Rayner, C. (2020). Children with autism in a sport and physical activity context: a collaborative autoethnography by two parents outlining their experiences. *Sport, Education & Society*, *25*(9), 1002-1014. <https://doi.org/10.1080/13573322.2019.1680535>

Nichols, C., Block, M. E., Bishop, J. C., & McIntire, B. (2019). Physical activity in young adults with autism spectrum disorder: Parental perceptions of barriers and facilitators. *Autism : the international journal of research and practice*, *23*(6), 1398-1407. <https://doi.org/10.1177/1362361318810221>

Obrusnikova, I., & Cavalier, A. R. (2011). Perceived Barriers and Facilitators of Participation in After-School Physical Activity by Children with Autism Spectrum Disorders. *Journal of developmental and physical disabilities*, *23*(3), 195-211. <https://doi.org/10.1007/s10882-010-9215-z>

Page, M. J., McKenzie, J. E., Bossuyt, P. M., Boutron, I., Hoffmann, T. C., Mulrow, C. D., ... & Moher, D. (2021). The PRISMA 2020 statement: an updated guideline for reporting systematic reviews. *BMJ*, 372, n71. https://doi.org/10.1136/bmj.n71

Papadopoulos, N. V., Whelan, M., Skouteris, H., Williams, K., McGinley, J., Shih, S. T. F., Emonson, C., Moss, S. A., Sivaratnam, C., Whitehouse, A. J. O., & Rinehart, N. J. (2020). An Examination of Parent-Reported Facilitators and Barriers to Organized Physical Activity Engagement for Youth With Neurodevelopmental Disorders, Physical, and Medical Conditions. *Frontiers in psychology*, *11*, 568723-568723. <https://doi.org/10.3389/fpsyg.2020.568723>

Pellerin, S., Wilson, W. J., & Haegele, J. A. (2022). The experiences of students with disabilities in self-contained physical education. *Sport, Education and Society*, *27*(1), 14-26. <https://doi.org/10.1080/13573322.2020.1817732>

Quesada, D., Ahmed, N. U., Fennie, K. P., Gollub, E. L. & Ibrahimou, B. (2018). A Review: Associations Between Attention-deficit/hyperactivity Disorder, Physical Activity, Medication Use, Eating Behaviors and Obesity in Children and Adolescents. Archives of psychiatric nursing, *32*(3), 495-504. <https://doi.org/10.1016/j.apnu.2018.01.006>

Ringer, N. (2020). Living with ADHD: A Meta-Synthesis Review of Qualitative Research on Children’s Experiences and Understanding of Their ADHD. *International Journal of Disability, Development, and Education*, *67*(2), 208–224. <https://doi.org/10.1080/1034912X.2019.1596226>

Rodriquez, J., Lanser, A., Jacobs, H. E., Smith, A., & Ganguly, S. (2022). When the Normative Is Formative: Parents’ Perceptions of the Impacts of Inclusive Sports Programs. *International Journal of Environmental Research and Public Health*, *19*(17), 10889. <https://doi.org/10.3390/ijerph191710889>

Sallis, J., Bauman, A., & Pratt, M. (1998). Environmental and policy interventions to promote physical activity. *American Journal of Preventive Medicine*, *15*(4), 379–397. <https://doi.org/10.1016/S0749-3797(98)00076-2>

Sandelowski, M. (2007). *Handbook for synthesizing qualitative research*. New York/London : Springer.

Shimoni, M. a., Engel-Yeger, B., & Tirosh, E. (2010). Participation in leisure activities among boys with attention deficit hyperactivity disorder. *RESEARCH IN DEVELOPMENTAL DISABILITIES*, *31*(6), 1234-1239. <https://doi.org/10.1016/j.ridd.2010.07.022>

Siu, A. F. Y., & Lo, J. W. K. (2020). Promising effect of a family rugby programme for children with ADHD: Promoting parent-child relationship and perceptual change on child's behaviors. *Complementary therapies in clinical practice*, *39*, 101135-101135. <https://doi.org/10.1016/j.ctcp.2020.101135>

Stanish, H., Curtin, C., Must, A., Phillips, S., Maslin, M., & Bandini, L. (2015). Enjoyment, barriers, and beliefs about physical activity in adolescents with and without autism spectrum disorder. *Adapted Physical Activity Quarterly*, *32*(4), 307-317. <https://doi.org/10.1123/APAQ.2015-0038>

Tandon, P. S., Sasser, T., Gonzalez, E. S., Whitlock, K. B., Christakis, D. A., & Stein, M. A. (2019). Physical activity, screen time, and sleep in children with ADHD. *Journal of physical activity & health*, *16*(6), 416-422. <https://doi.org/10.1123/jpah.2018-0215>

Thoren, A., Quennerstedt, M., & Maivorsdotter, N. (2021). What physical education becomes when pupils with neurodevelopmental disorders are integrated: a transactional understanding. *Physical Education and Sport Pedagogy*, *26*(6), 578-592. <https://doi.org/10.1080/17408989.2020.1834525>

Vargas, T. M., Flores, M. M., & Beyer, R. (2015). Coaches’ Perceptions and Proposed Solutions for Challenging Behaviors: Implications for Athletes with Hidden Disabilities. *International Journal of Sports Science & Coaching*, *10*(5), 783–796. <https://doi.org/10.1260/1747-9541.10.5.783>

Vargas, T. M., Flores, M. M., Beyer, R., & Weaver, S. M. (2019). Parents’ Perceptions of Coaching Behaviors toward Their Child with a Hidden Disability in Recreational Youth Sports. *The Physical Educator*, *76*(3), 661–675. <https://doi.org/10.18666/TPE-2019-V76-I3-8814>

World Health Organisation, WHO (2019). Attention Deficit Hyperactivity Disorder (ADHD). Downloaded (20230809) from <https://iris.who.int/bitstream/handle/10665/364129/WHOEMMNH214E-eng.pdf?sequence=1&isAllowed=y>

Yessick, A. B., Haegele, J. A., & Zhu, X. (2020). Exploring the Experiences of Children with ASD in Self-contained Physical Education: a Modified Scrapbooking Study. *Advances in Neurodevelopmental Disorders*, 4(1), 1–12. <https://doi.org/10.1007/s41252-019-00139-5>

Zeidan, J., Fombonne, E., Scorah, J., Ibrahim, A., Durkin, M.S., Saxena, S. et al. (2022). Global prevalence of autism: A systematic review update. Autism research,*15*(5), 778-790. <https://doi.org/10.1002/aur.2696>

1. 5-8% of children are diagnosed with ADHD (WHO, 2019) and about 1 % have an ASD diagnosis (Zeidan et al., 2022). Boys are more often diagnosed than girls. [↑](#footnote-ref-1)
2. Children are all humans up to the age of 18, according to the United Nation’s definition; however, when a report specifically includes adolescents (ages 10-19 according to the World Health Organisation’s definition) this term will also be used. [↑](#footnote-ref-2)
3. Adapted forms of PE and sports are adjusted to the person with a disability. Adapted forms of sport/PE can take place in a general PE class/sport team with accommodations or, in small groups, or in an individual setting, depending on the needs of the child. Self-contained physical education is an alternative class specifically designed for students with disabilities who risk not to learn satisfactory within integrated physical education. Activities are usually preformed in smaller groups and with specific adaptions. [↑](#footnote-ref-3)
4. Search for literature published 2010-2021: SPORT DISCUS (13-02-22), ERIC (15-02-22), EDUCATION DATABASE (15-02-22), SCOPUS (16-02-22), WEB OF SCIENCE (sport science och Education special, 16-02-22), GU search (11-03-22). Search for literature published 2022-2023: SPORT DISCUS (12-04-24), ERIC (12-04-24), EDUCATION DATABASE (12-04-24), SCOPUS (12-04-24), WEB OF SCIENCE (sport science och Education special, 06-05-21), GU search (07-06-24). Reference lists included in the sample have been scanned for additional references. [↑](#footnote-ref-4)
5. Neuro developmental disorders such as ADHD and ASD are sometime referred to as “hidden disabilities” since they are disabilities that do not show from an outside perspective. [↑](#footnote-ref-5)
6. For the clarity of this literature review, I have used the concepts LTPA and PE to categorise activities in table 1. The categorisation is based on descriptions of activities in the included articles. In case an article contain result on other activities that cannot be categorised as organised physical activity (i.e. playing computer games or waking the dog) these results have not been included in the synthesis. [↑](#footnote-ref-6)
